# Rational Design, Synthesis, and Biological Evaluation of New Substituted Dihydropyrimidinones as Antibacterial Agents and *β*‐Lactamase Inhibitors

**DOI:** 10.1111/cbdd.70374

**Published:** 2026-08-04

**Authors:** Ahmed M. Soliman, Heba Abdelrasheed Allam, Walaa R. Mahmoud, Bassem H. Naguib

**Affiliations:** ^1^ Pharmaceutical Chemistry Department, Faculty of Pharmacy The British University in Egypt El‐Shorouk Egypt; ^2^ Pharmaceutical Chemistry Department, Faculty of Pharmacy Cairo University Cairo Egypt

**Keywords:** antimicrobial assay, *β*‐lactamase inhibitor antibacterial agents, dihydropyrimidinones

## Abstract

A series of new substituted 1,6‐dihydropyrimidinones was designed, synthesized, and biologically evaluated as potential antibacterial candidates and *β*‐lactamase enzyme inhibitors. All the synthesized compounds were tested for their antibacterial activity against *Staphylococcus aureus, Bacillus subtilis* as a gram‐positive species whereas *Pseudomonas aeruginosa* as a gram‐negative species was utilized. Most of the compounds exhibited moderate antibacterial activity compared to the reference drug amoxicillin. Furthermore, clinical antibacterial tests were conducted on *β*‐lactamase resistant strains, including *Acinetobacter baumannii, Bacillus subtilis*, and *Staphylococcus aureus*. Results indicated that the candidate compounds possess potentiation effect, indicating their role as effective *β*‐lactamase enzyme inhibitors. Whereas *in‐vitro* assay performed on *β*‐lactamase enzymes revealed that compounds **3d**, **3i**, and **7b** (IC_50_ = 0.758, 0.400, and 0.524 nM) have lower IC_50_ values compared to clavulanic acid (IC_50_ = 0.934 nM). These findings suggested that compounds **3d**, **3i**, and **7b** are considered as promising hits for further exploration of potent and selective *β*‐lactamase inhibitors.

## Graphical abstract

Novel 1,6‐dihydropyrimidinones act as antibacterial agents and beta‐lactamase inhibitors. Compounds **3d**, **3i**, and **7b** demonstrated enhanced beta‐lactamase inhibition relative to the standard reference, clavulanic acid, demonstrating significant potentiation effects to restore antibiotic efficacy and combat resistant bacterial strains.

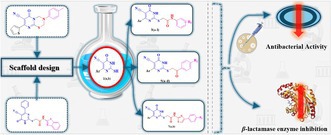

## 1 Introduction

Health care‐associated infections (HAIs) impact patients and health systems, resulting in significant suffering, escalating health care expenses, and obstructing the application of high‐quality care for all. HAIs are frequently challenging to manage and significantly contribute to antimicrobial resistance (AMR; WHO 2024).

WHO has approved five essential strategic action plans for tackling antimicrobial resistance (AMR) that include (1) enhancing the awareness of antimicrobial resistance; (2) strengthening the knowledge through surveillance and research in combating infection via control measures; (3) executing effective sanitation, hygiene, and infection prevention measures; (4) optimizing the utilization of antimicrobials in human and animal health; and (5) promoting sustainable investment in new medicines, diagnostic tools, and vaccines (WHO 2022).

The *β*‐lactam antibiotics contain a ring structure that is similar to the D‐alanine of the pentapeptide in *N*‐acetylmuramic acid (NAM). This similarity prompts the penicillin‐binding proteins (PBPs) to utilize *β*‐lactam as a structural component in substitute of the NAM pentapeptide. The acylation of both enzymes and *β*‐lactam antibiotics is irreversible, rendering the enzyme incapable of catalysing any transpeptidation of the cell wall (Zapun et al. 2008).

*β‐*Lactamase enzymes were among the most prevalent mechanisms of resistance to *β‐*Lactam antibiotics. Two strategies have been employed to tackle *β‐*lactamase‐mediated resistance to *β‐*lactam antibiotics: the modification of the antibiotic's structure to prevent it from functioning as a substrate for the enzyme, and the inhibition of the enzyme with a compound that is structurally related to the *β‐*lactam substrate. Recently, the utilization of *β‐*lactamase inhibitors has been the most successful approach, as the rapid evolution of resistant pathogens has led to the eventual emergence of *β‐*lactamases that can hydrolyze the modified *β‐*lactams that have been developed (Williams 1999).

AMR occurs when microbes evolve mechanisms that protect them from the effects of antimicrobials. All classes of microbes can evolve resistance to the point that one or more drugs used to fight them are no longer effective (Magiorakos et al. 2012). Microbes resistant to multiple antimicrobials are called multidrug resistant (MDR) and are sometimes referred to as superbugs. Although antimicrobial resistance is a naturally occurring process, it often occurs because of the improper usage of the drugs and management of the infections. Different microorganisms can exhibit resistance to antimicrobials through a variety of mechanisms (WHO 2023), among which is encoding genes to produce enzymes that destroy antibiotics such as *β*‐Lactamase enzyme, which attacks the *β*‐Lactam ring in antibiotics such as penicillins and cephalosporins, rendering them inactive.

Other mechanisms include the efflux pumps produced by the microorganisms in the cell membrane to extrude the antimicrobials from the cell before exerting their action. Additionally, microorganisms could acquire resistant genes from other microorganisms through different routes such as transformation, conjugation, or transduction (McManus 1997).

Resistance can occur by various mechanisms due to *β*‐lactam drugs' extensive use. The production of *β*‐Lactamase enzymes is a milestone for *β*‐lactam drugs' resistance. Gram‐positive and gram‐negative bacteria produce the mentioned enzymes, leading to the hydrolysis of the *β*‐lactam amide moiety (Bush 2018).

### 1.1 In Gram‐Positive Bacteria

The initial mechanism of resistance to *β*‐lactam antibiotics involved the acquisition of penicillin‐binding proteins (PBPs) with lower affinity for *β*‐lactam drugs. This form of resistance was first observed in clinical isolates of *Streptococcus pneumoniae* that were resistant to penicillin (Bullen and Hansman 1975; Dowson et al. 1989) and in methicillin‐resistant *S. aureus* (MRSA; Hartman and Tomasz 1984). Additionally, the excessive use of *β*‐lactam drugs for the treatment of nosocomial streptococcal infections led to the overproduction of penicillinases or *β*‐lactamases as collateral damage (Rammelkamp and Maxon 1942).

### 1.2 In Gram‐Negative Bacteria

**FIGURE 1:**
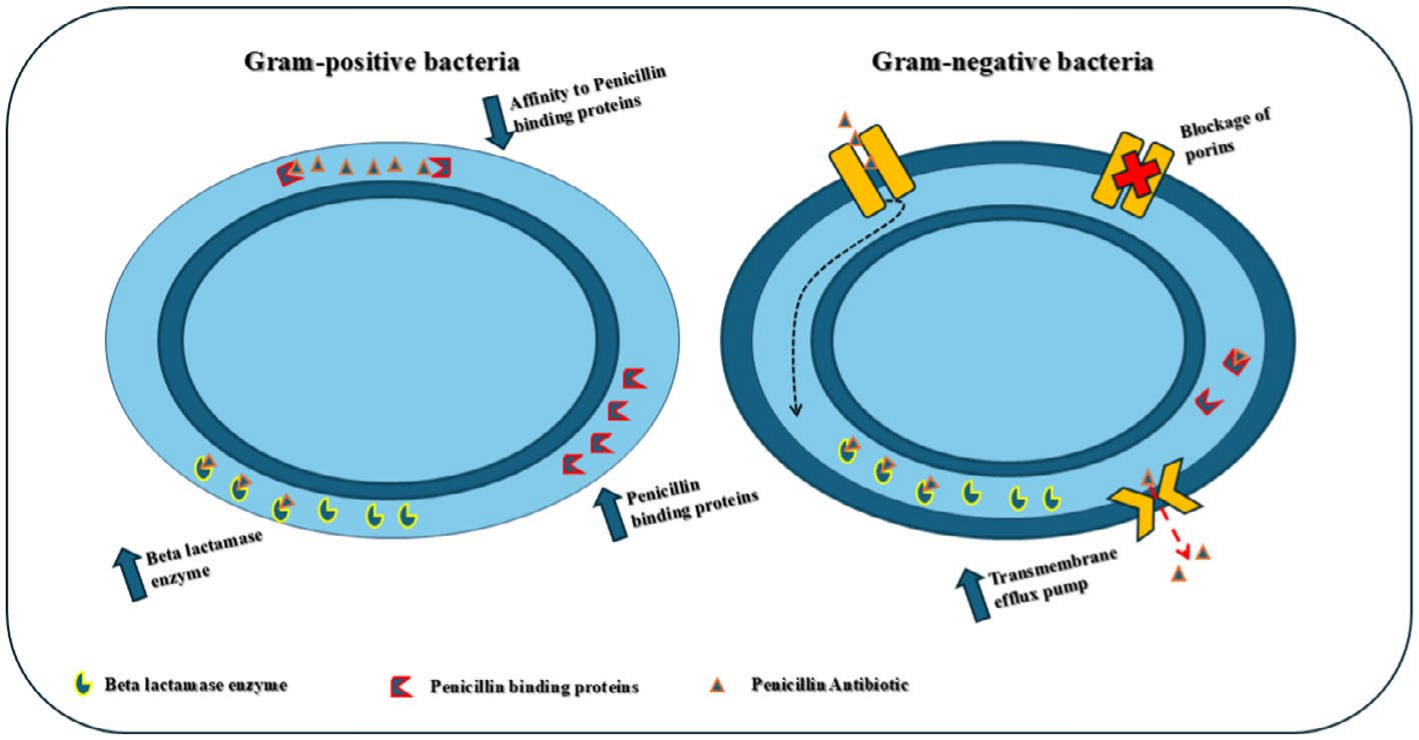
The effect of *β*‐lactamase enzyme in both gram‐positive and gram‐negative bacteria.

*β*‐lactamase enzymes played a crucial role in gram‐negative bacteria resistance to *β*‐lactam antibiotics that has become more prevalent, particularly in enteric bacteria (such as *Enterobacter* species) and *Pseudomonas aeruginosa* . In these organisms, resistance is often mediated by the production of inducible chromosomal *β*‐lactamases. These enzymes are encoded by chromosomal genes and can be upregulated in response to the presence of *β*‐lactam antibiotics, leading to increased hydrolysis of these drugs and subsequent resistance (Labia et al. 1977; Matthew and Harris 1976). Furthermore, the excessive use of *β*‐lactam antibiotics leads to loss of the outer membrane porin and activation of transmembrane efflux pumps (Figure 1).

Interestingly, extensive research has been directed towards the identification of substituted dihydropyrimidinone (DHPMs) as antibacterial agents (Chitra et al. 2010; Die et al. 1835; Hassan et al. 2015; Taher and Abou‐Seri 2012). It has been found that different substituted DHPMs have been reported to have promising antibacterial activity. **Compound I** showed a higher inhibition zone against *B. subtilis* (26 cm) compared to ampicillin and Cefotaxime (22 cm and 25 cm, respectively; Hassan et al. 2015). On the other hand, **compound II** demonstrated the same inhibitory activity to the growth of *E. coli* as ampicillin (MIC = 125 μg/mL; Rizk et al. 2018).

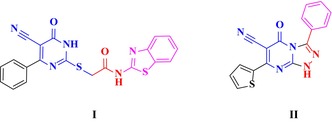

**FIGURE 2:**
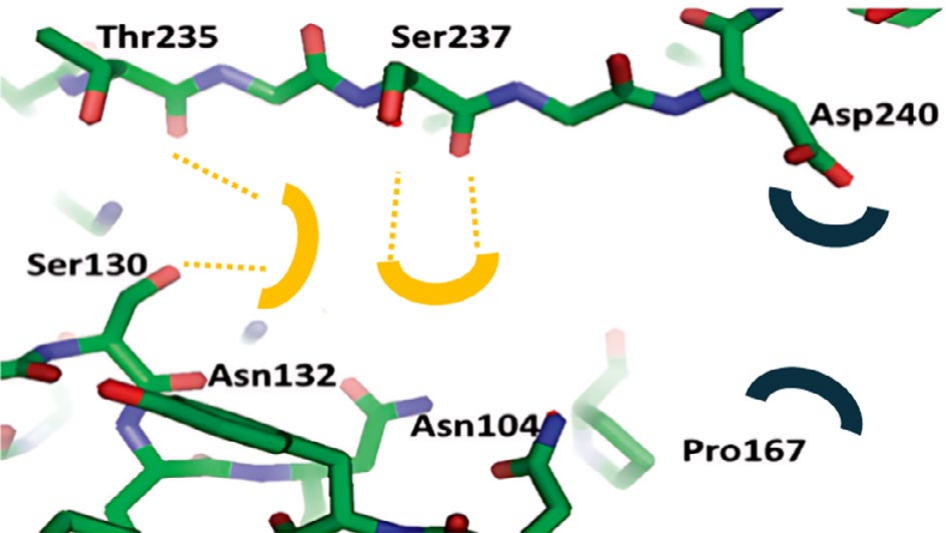
The binding mode of *β‐*lactamase inhibitors (Nichols et al. 2012).

At this point, an overview of the pharmacophoric features of different *β*‐lactamase inhibitors with the binding site is essential in designing the target compounds. *β*‐lactamase inhibitors investigation mentioned two key regions in the binding site that could be targeted to strengthen the interaction (Figure 2). The first region is a nonpolar binding surface around Pro167 while the other region involves Asp240 that presents an opportunity for favorable electrostatic interactions (Blue color). Designing and developing compounds to interact with these two “hot spots” has resulted in the discovery of several new high‐affinity noncovalent inhibitors. Additionally, hydrogen bonds formation between Thr235, Ser237, and Ser130 and the target compounds (Orange color; Mohamed et al. 2011; Nichols et al. 2012).

Literature review reveals that substituted pyrimidines **III** and **IV** exhibit significant inhibitory activity against the IMP‐1 *β*‐lactamase enzyme. At a test concentration of 10 μM, these compounds demonstrated inhibitory concentrations of 38 μM and 29 μM, respectively, showing promising potency relative to the reference inhibitor 12 μM under identical assay conditions (Hussein et al. 2012).

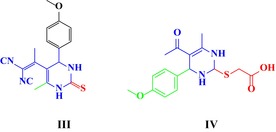

Based on the prior facts, and with the aim of creating new antibacterial agents and inhibitors for *β*‐lactamase enzymes, the DHPM ring was selected as the core structure for the new target compounds. This ring will function as the heterocyclic moiety that will compete for binding in the different binding sites. Given that most reported candidates containing DHPM are substituted at positions 2 and 4 of the DHPM scaffold, the objective of this study is to design and synthesize a new series of 2,4‐disubstituted DHPM derivatives.

**FIGURE 3:**
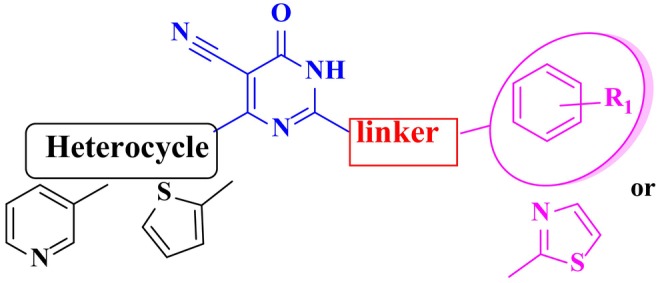
Development strategy of the target compounds.

The development strategy of the proposed scaffold is to modify the side chain at position **2**. This can be modified by adding a substituted phenyl ring as in compounds **3a‐*l*** and **5a‐d** or substituted thiazolyl moieties as in compounds **7a,b**. In compounds **3a‐l**, the phenyl ring is either unsubstituted or substituted by electron donating or electron withdrawing groups to study the electronic effect of these substituents on activity. While in compounds **5a‐d**, the phenyl ring is either substituted by methyl as an electron donating group or by a bromo group as an electron‐withdrawing group to study the electronic effect of these substituents on activity. Similarly, compounds **7a,b** contain a thiazole ring *para* substituted with either methyl or a bromo group. This setup is used to explore how these specific substitutions affect the activity. These substituents are attached to the DHPM ring through different linkers such as thioacetamide as in compounds **3a‐l** and **7a,b** and thioacetyl as in compounds **5a‐d (**Figure 3). Concerning position 4 of the DHPM ring, it was substituted with either a pyridine or thiophene heterocycle.

## 2 Experimental

### 2.1 Chemistry

Melting points were measured with a Stuart SMP 30 (uncorrected). IR spectra were recorded on a Shimadzu FT‐IR 8400S. ^1^H and ^13^C NMR spectra (400/100 MHz, DMSO‐*d*_*6*_) were obtained using a Bruker Ascend 400/R at Cairo University and Mansura University, referenced to TMS. Coupling constants (*J*) are in Hz (Figures S1–S36). Elemental analysis was performed with a Thermo Scientific Flash 2000, and mass spectra with a Thermo Scientific GCMS ISQ at Al‐Azhar University. TLC on Merck silica gel plates monitored reactions and purity. All reagents and solvents were purified and dried by standard methods. Compounds **1 (a,b)** (Chhillar et al. 2006; Ramiz et al. 2011), **2 (a‐f)** (Albohy et al. 2024; Tong et al. 2012), **4 (a,b)** (Amblard et al. 2013), and **6 (a,b)** (Hassan et al. 2012; Sharma et al. 2019) were prepared and characterized through reported methods.

#### 2.1.1 General Procedure for Preparation of Target Compounds 3 (a‐l), 5 (a‐d) and 7 (a,b)

A mixture of the appropriate compound **1 (a,b)** (0.002 mol) and triethylamine (0.228 g, 0.003 mol) was refluxed in (200 mL) until complete dissolution. Potassium carbonate (0.414 g, 0.003 mol), potassium iodide (0.332 g, 0.002 mol), and the compound **2 (a–f)**, substituted phenacyl bromide **4 (a,b)**, or compound **6 (a,b)** (0.003 mol) were then added. The reaction mixture was refluxed for 20–24 h with progress monitored by TLC. The precipitate was collected by filtration, washed, dissolved in hot water, and neutralized with glacial acetic acid and then filtered. The resulting solid was washed with water, dried, and recrystallized from dimethyl formamide/water.

##### 2.1.1.1 2‐[(5‐Cyano‐6‐Oxo‐4‐(Pyridin‐3‐Yl)‐1,6‐Dihydropyrimidin‐2‐Yl)thio]‐*N*‐Phenylacetamide (3a)

Pale yellow powder (yield 82%), m.p. 240°C–242°C; IR (KBr) (cm^−1^): 3417–3340 (2NH), 3062–3028 (CH aromatic), 2947–2908 (CH aliphatic), 2222 (C≡N), 1678 and 1631 (C=O), 1597–1575 (C=N), 1492–1473 (C=C). ^1^H NMR (400 MHz, DMSO‐*d*_*6*_) δ 4.30 (s, 2H, ‐CH_2_‐), 5.81 (s, 1H, ‐NH‐, D_2_O exchangeable), 7.03 (t, *J* = 7.4 Hz, 1H, Ar‐H), 7.27 (t, *J* = 7.7 Hz, 2H, Ar‐H), 7.55 (d, *J* = 8.0 Hz, 2H, Ar‐H), 7.67–7.76 (dd, *J* = 7.5, 5.4, 7.5 Hz, 1H, pyridine H), 8.64 (d, *J* = 8.3 Hz, 1H, pyridine H), 8.86 (d, *J* = 5.0 Hz, 1H, pyridine H), 9.32 (s, 1H, pyridine H), 10.64 (s, 1H, ‐NH‐, D_2_O exchangeable); ^13^C NMR (100 MHz, DMSO‐ *d*_*6*_) δ = 36.15 (‐CH_2_‐), 95.02, 115.74 (C≡N), 119.55, 123.90, 125.36, 129.18, 132.73, 139.23, 140.52, 146.69, 149.25, 160.92, 164.00, 165.67 (C=O amide), 167.20 (C=O amide); MS: m/z (%) 363.35 (M^+^, 60.19), 174.09 (100) Anal. Calcd. for C_18_H_13_N_5_O_2_S (363.40): C, 59.49; H, 3.61; N, 19.27 found: C, 59.70; H, 3.83; N, 19.45.

##### 2.1.1.2 2‐[(5‐Cyano‐6‐Oxo‐4‐(Pyridin‐3‐Yl)‐1,6‐Dihydropyrimidin‐2‐Yl)thio]‐*N*‐(P‐Tolyl)acetamide (3b)

Buff powder (yield 84%), m.p. 230°C–232°C; IR (KBr) (cm^−1^): 3398–3348 (2NH), 3101–3047 (CH aromatic), 2958–2927 (CH aliphatic), 2222 (C≡N), 1678 and 1641 (C=O), 1604–1584 (C=N), 1516–1477 (C=C); ^**1**^ **H NMR** (400 MHz, DMSO‐*d*_*6*_) δ 2.24 (s, 3H, ‐CH_3_), 4.27 (s, 2H, ‐CH_2_‐), 5.37 (s, 1H, ‐NH‐, D_2_O exchangeable), 7.08 (d, *J* = 8.1 Hz, 2H, Ar‐H), 7.43 (d, *J* = 8.4 Hz, 2H, Ar‐H), 7.69 (dd, *J* = 8.1, 5.1 Hz, 1H, pyridine H), 8.58 (d, *J* = 8.2 Hz, 1H, pyridine H), 8.85 (d, *J* = 5.0 Hz, 1H, pyridine H), 9.29 (s, 1H, pyridine H), 10.47 (s, 1H, ‐NH‐, D_2_O exchangeable); ^13^C NMR (100 MHz, DMSO‐*d*_*6*_) δ 20.91 (‐CH_3_), 36.12 (‐CH_2_‐), 95.00, 115.75 (C≡N), 119.55, 125.36, 129.55, 132.71, 132.83, 136.73, 140.49, 146.70, 149.28, 160.90, 163.99, 165.38 (C=O amide), 167.21 (C=O amide); MS: m/z (%) 377.01 (M^+^, 11), 360.37 (100); Anal. Calcd. for C_19_H_15_N_5_O_2_S (377.42): C, 60.47; H, 4.01; N, 18.56; found: C, 60.71; H, 4.20; N, 18.79.

##### 2.1.1.3 2‐[(5‐Cyano‐6‐Oxo‐4‐(Pyridin‐3‐Yl)‐1,6‐Dihydropyrimidin‐2‐Yl)thio]‐*N*‐(4‐Isopropylphenyl)acetamide (3c)

Brown powder (yield 87%), m.p. 237°C–240°C; IR (KBr) (cm^−1^): 3406–3348 (2NH), 3059–3032 (CH aromatic), 2920–2854 (CH aliphatic), 2218 (C≡N), 1670 and 1631 (C=O), 1604–1592 (C=N), 1516–1481 (C=C); ^1^H NMR (400 MHz, DMSO‐*d*_*6*_) δ 1.18 (d, *J* = 6.9 Hz, 6H, CH‐C_2_ H_6_), 2.84 (m, *J* = 6.8 Hz, 1H, ‐CH‐C_2_H_6_), 4.26 (s, 2H, ‐CH_2_‐), 4.44 (s, 1H, ‐NH‐, D_2_O exchangeable), 7.15 (d, *J* = 8.23 Hz, 2H, Ar‐H), 7.44 (d, *J* = 8.3 Hz, 2H, Ar‐H), 7.59 (dd, *J* = 5.0, 8.0 Hz, 1H, pyridine H), 8.46 (d, *J* = 8.16 Hz, 1H, pyridine H), 8.81 (d, *J* = 4.87 Hz, 1H, pyridine H), 9.22 (s, 1H, pyridine H), 10.37 (s, 1H, ‐NH‐, D_2_O exchangeable). ^13^C NMR (100 MHz, DMSO‐*d*_*6*_) δ 24.40 (2CH_3_), 33.31 (‐CH‐), 36.12 (‐CH_2_‐), 94.81, 115.82 (C≡N), 119.73, 124.78, 126.89, 132.30, 136.95, 139.14, 144.03, 147.79, 150.50, 160.98, 164.53165.37 (C=O amide), 167.06 (C=O amide); MS: m/z (%) 405.44 (M^+^, 9.36), 313.58 (100); Anal. Calcd. for C_21_H_19_N_5_O_2_S (405.48): C, 62.21; H, 4.72; N, 17.27; found: C, 61.98; H, 4.80; N, 17.49.

##### 2.1.1.4 2‐[(5‐Cyano‐6‐Oxo‐4‐(Pyridin‐3‐Yl)‐1,6‐Dihydropyrimidin‐2‐Yl)thio]‐*N*‐(4‐Fluorophenyl)acetamide (3d)

Yellow powder (yield 77%), m.p. 228°C–230°C; IR (KBr) (cm^−1^): 3244–3213 (2NH), 3147–3056 (CH aromatic), 2939–2897 (CH aliphatic), 2222 (C≡N), 1693 and 1658 (C=O), 1631–1615 (C=N), 1560–1535 (C=C); ^1^H NMR (400 MHz, DMSO‐*d*_*6*_) δ 4.19 (s, 2H, ‐CH_2_‐), 7.09–7.19 (m, 2H, Ar‐H), 7.43 (dd, *J* = 4.8, 7.9 Hz, 1H, pyridine H), 7.54 (dd, *J* = 4.9, 8.86 Hz, 2H, Ar‐H), 8.22 (d, *J* = 8.0 Hz, 1H, pyridine H), 8.72 (d, *J* = 3.1 Hz, 1H, pyridine H), 9.07 (s, 1H, pyridine H), 10.41 (s, 1H, ‐NH‐); ^13^C NMR (100 MHz, DMSO‐*d*_*6*_) δ 36.00 (‐CH_2_‐), 94.23, 115.70 (C≡N), 116.20, 121.37, 121.45, 123.82, 131.64, 135.49, 135.49, 135.51, 136.73, 149.49, 152.43, 157.36, 159.74, 161.66, 165.41, 165.70 (C=O amide), 167.09 (C=O amide); MS: m/z (%) 381.16 (M^+^, 49.93), 152.90 (100). Anal. Calcd. for C_18_H_12_FN_5_O_2_S (381.39): C, 56.69; H, 3.17; N, 18.36; found: C, 56.82; H, 3.40; N, 18.61.

##### 2.1.1.5 2‐[(5‐Cyano‐6‐Oxo‐4‐(Pyridin‐3‐Yl)‐1,6‐Dihydropyrimidin‐2‐Yl)thio]‐*N*‐(3,4‐Dichlorophenyl)acetamide (3e)

White powder (yield 83%), m.p. 227°C–230°C; IR (KBr) (cm^−1^): 3581–3441 (2NH), 3093–3051 (CH aromatic), 2993–2935 (CH aliphatic), 2222 (C≡N), 1678 and 1628 (C=O), 1587–1567 (C=N), 1516–1460 (C=C); ^1^H NMR (400 MHz, DMSO‐*d*_*6*_) δ 4.36 (s, 2H, ‐CH_2_‐), 4.48 (s, 1H, ‐NH‐), 7.26 (t, 1H, Ar‐H), 7.42 (t, *J* = 7.5 Hz, 1H, Ar‐H), 7.50 (d, *J* = 8.1 Hz, 1H, Ar‐H), 7.66 (dd, *J* = 5.0, 8.0 Hz, 1H, pyridine H), 8.49 (d, *J* = 9.74 Hz, 1H, pyridine H), 8.80 (d, *J* = 4.96 Hz, 1H, pyridine H), 9.20 (s, 1H, pyridineH), 10.11 (s, 1H, ‐NH‐); ^13^C NMR (100 MHz, DMSO‐*d*_*6*_) δ 35.52 (‐CH_2_‐), 95.02, 115.74 (C≡N), 124.86, 125.21, 125.61, 127.32, 128.36, 132.29, 132.61, 136.91, 140.06, 147.06, 149.59160.99, 164.25, 166.48 (C=O amide), 166.97 (C=O amide); MS: m/z (%) 433.90 (M^+^+2, 31.70), 431.26 (M^+^, 38.81), 379.38 (100); Anal. Calcd. for C_18_H_11_Cl_2_N_5_O_2_S (431.28): C, 50.01; H, 2.57; N, 16.20; found: C, 50.27; H, 2.68; N, 16.45.

##### 2.1.1.6 *N*‐(4‐Chlorophenyl)‐2‐[(5‐Cyano‐6‐Oxo‐4‐(Pyridin‐3‐Yl)‐1,6‐Dihydropyrimidin‐2‐Yl)thio]Acetamide (3f)

Orange powder (yield 73%), m.p. 238°C–240°C; IR (KBr) (cm^−1^): 3344–3410 (2NH), 3101–3059 (CH aromatic), 2924–2850 (CH aliphatic), 2222 (C≡N), 1678 and 1628 (C=O), 1600.92–1584 (C=N), 1535–1492 (C=C); ^1^H NMR (400 MHz, DMSO‐*d*_*6*_) δ 4.27 (s, 2H, ‐CH_2_‐), 4.38 (s, 1H, ‐NH‐), 7.33 (d, *J* = 8.84 Hz, 2H, Ar‐H), 7.58 (d, *J* = 8.8 Hz, 2H, Ar‐H), 7.63 (dd, *J* = 5.0, 8.0 Hz, 1H, pyridine H), 8.49 (d, *J* = 8.1 Hz, 1H, pyridine H), 8.81 (d, *J* = 3.5 Hz, 1H, pyridine H), 9.22 (s, 1H, pyridine H), 10.73 (s, 1H, ‐NH‐); ^13^C NMR (101 MHz, DMSO‐*d*_*6*_) δ 36.09 (‐CH_2_‐), 95.09, 115.69 (C≡N), 121.03, 125.62, 127.38, 129.04, 132.86, 138.21, 141.13, 146.18, 148.68, 160.88, 163.72, 165.91 (C=O amide), 167.20 (C=O amide); MS: m/z (%) 398.93 (M^+^+2, 36.30), 396.60 (M^+^, 63.88), 224.45 (100); Anal. Calcd. for C_18_H_12_ClN_5_O_2_S (397.84): C, 54.34; H, 3.04; N, 17.60; found: C, 54.67; H, 3.21; N, 17.82.

##### 2.1.1.7 2‐[(5‐Cyano‐6‐Oxo‐4‐(Thiophen‐2‐Yl)‐1,6‐Dihydropyrimidin‐2‐Yl)thio]‐*N*‐Phenylacetamide (3 g)

White powder (yield 75%), m.p. 238°C–240°C; IR (KBr) (cm^−1^): 3329–3197 (2NH), 3101–3020 (CH aromatic), 2931–2854 (CH aliphatic), 2222 (C≡N), 1689 and 1662 (C=O), 1647.92–1626 (C=N), 1601–1546 (C=C); ^1^H NMR (400 MHz, DMSO‐*d*_*6*_) δ 4.23 (s, 2H, ‐CH_2_‐), 7.05 (t, *J* = 7.3 Hz, 1H, Ar‐H), 7.27 (t, *J* = 4.4 Hz, 1H, thiophene H), 7.31 (t, *J* = 7.8 Hz, 1H, Ar‐H, 1H), 7.58 (d, *J* = 7.9 Hz, 1H, Ar‐H), 7.99 (d, *J* = 4.9 Hz, 1H, thiophene H), 8.25 (d, *J* = 3.7 Hz, 1H, thiophene H), 10.41 (s, 1H, ‐NH‐, D_2_O exchangeable); ^13^C NMR (100 MHz, DMSO‐*d*_*6*_) δ 35.88 (‐CH_2_‐), 88.57, 116.70 (C≡N), 119.75, 123.98, 129.24, 129.77, 132.29, 135.19, 139.28, 139.55, 159.20, 161.52, 165.29 (C=O amide), 165.79 (C=O amide); MS: m/z (%) 368.28 (M^+^, 3.95), 324.20 (100); Anal. Calcd. for C_17_H_12_N_4_O_2_S_2_ (368.43): C, 55.42; H, 3.28; N, 15.21; found: C, 55.34; H, 3.31; N, 15.23.

##### 2.1.1.8 2‐[(5‐Cyano‐6‐Oxo‐4‐(Thiophen‐2‐Yl)‐1,6‐Dihydropyrimidin‐2‐Yl)thio]‐*N*‐(p‐Tolyl)acetamide (3 h)

White powder (yield 74%), m.p. 233°C–235°C; IR (KBr) (cm^−1^): 3375–3275 (2NH), 3113–3047 (CH aromatic), 2985–2927 (CH aliphatic), 2222 (C≡N), 1684 and 1654 (C=O), 1597–1567 (C=N), 1527–1477 (C=C); ^1^H NMR (400 MHz, DMSO‐*d*_*6*_) δ 2.25 (s, 3H, ‐CH_3_), 4.22 (s, 2H, ‐CH_2_‐), 7.11 (d, *J* = 8.1 Hz, 2H, Ar‐H), 7.29 (t, 1H, thiophene H), 7.46 (d, *J* = 8.3 Hz, 2H, Ar‐H), 8.01 (d, *J* = 5.0 Hz, 1H, thiophene H), 8.25 (d, *J* = 2.7 Hz, 1H, thiophene H), 10.30 (s, 1H, ‐NH‐), 13.76 (s, 1H, ‐NH‐); ^13^C NMR (100 MHz, DMSO‐*d*_*6*_) δ 20.90 (‐CH_3_), 35.90 (‐CH_2_‐), 88.62, 116.68 (C≡N), 119.75, 129.60, 129.77, 132.30, 132.87, 135.24, 136.82, 139.59, 159.18, 161.42, 164.98 (C=O amide), 165.75 (C=O amide); MS: m/z (%) 384.66 (M^+^, 41.47), 152.21 (100); Anal. Calcd. for C_18_H_14_N_4_O_2_S_2_ (382.46): C, 56.53; H, 3.69; N, 14.65; found: C, 56.45; H, 3.65; N, 14.71.

##### 2.1.1.9 2‐([(5‐Cyano‐6‐Oxo‐4‐(Thiophen‐2‐Yl)‐1,6‐Dihydropyrimidin‐2‐Yl)]thio)‐*N*‐(4‐Isopropylphenyl)Acetamide (3i)

Orange powder (yield 79%), m.p. 232°C–235°C; IR (KBr) (cm^−1^): 3302–3190 (2NH), 3101–3035 (CH aromatic), 2962–2931 (CH aliphatic), 2222 (C≡N), 1658 and 1620 (C=O), 1597 (C=N), 1527–1477 (C=C); ^1^H NMR (400 MHz, DMSO‐*d*_*6*_) δ 1.17 (d, *J* = 6.8 Hz, 6H, ‐CH‐C_2_ H_6_), 2.77–2.89 (m, 1H, ‐CH‐C_2_H_6_), 4.22 (s, 2H, ‐CH_2_‐), 7.18 (d, *J* = 8.4 Hz, 2H, Ar‐H), 7.29 (t, 1H, thiophene H), 7.48 (d, *J* = 8.4 Hz, 2H, Ar‐H), 8.01 (d, *J* = 5.0 Hz, 1H, thiophene H), 8.26 (d, *J* = 3.8 Hz, 1H, thiophene H), 10.31 (s, 1H, ‐NH‐), 13.65 (s, 1H, ‐NH‐); ^13^C NMR (100 MHz, DMSO‐*d*_*6*_) δ 24.37 (2CH_3_), 33.30 (CH), 35.83 (‐CH_2_‐), 88.54, 116.70 (C≡N), 119.91, 126.92, 129.79, 132.30, 135.22, 137.01, 139.59, 144.07, 159.19, 161.52, 165.05 (C=O amide), 165.80 (C=O amide); MS: m/z (%) 412.46 (M^+^, 28.44), 149.13 (100); Anal. Calcd. for C_20_H_18_N_4_O_2_S_2_ (410.51): C, 58.52; H, 4.42; N, 13.65; found: C, 58.50; H, 4.45; N, 13.81.

##### 2.1.1.10 2‐[(5‐Cyano‐6‐Oxo‐4‐(Thiophen‐2‐Yl)‐1,6‐Dihydropyrimidin‐2‐Yl)thio]‐*N*‐(4‐Fluorophenyl)acetamide (3j)

Orange powder (yield 79%), m.p. 230°C–233°C; IR (KBr) (cm^−1^): 3325–3242 (2NH), 3151–3101 (CH aromatic), 2997–2935 (CH aliphatic), 2214 (C≡N), 1708 and 1658 (C=O), 1550–1534 (C=N), 1535–1477 (C=C); ^1^H NMR (400 MHz, DMSO‐*d*_*6*_) δ 3.89 (s, 2H, ‐CH_2_‐),7.14 (t, *J* = 8.8 Hz, 2H, Ar‐H), 7.21 (t, 1H, thiophene H), 7.61 (dd, J = 5.0, 8.9 Hz, 2H, Ar‐H), 7.79 (d, J = 5.0 Hz, 1H, thiophene H), 8.11 (d, J = 3.8 Hz, 1H, thiophene H), 10.84 (s, 1H, ‐NH‐).^13^C NMR (100 MHz, DMSO‐*d*_*6*_) δ 35.50 (‐CH_2_‐), 86.30, 115.68 (C≡N), 115.91, 120.09, 121.03, 121.11, 128.81, 129.31, 131.50, 135.97, 135.99, 141.77, 157.17, 159.31, 159.55, 167.57, 169.38 (C=O amide), 171.13 (C=O amide); MS: m/z (%) 388.87 (M^+^, 24.05), 247.63 (100); Anal. Calcd. for C_17_H_11_FN_4_O_2_S_2_ (386.42): C, 52.84; H, 2.87; N, 14.50; found: C, 52.83; H, 2.91; N, 14.69.

##### 2.1.1.11 2‐[(5‐Cyano‐6‐Oxo‐4‐(Thiophen‐2‐Yl)‐1,6‐Dihydropyrimidin‐2‐Yl)thio]‐*N*‐(3,4‐Dichlorophenyl)acetamide (3 k)

Orange powder (yield 88%), m.p. 239°C–240°C; IR (KBr) (cm^−1^): 3290–3159 (2NH), 3082–3039 (CH aromatic), 2931–2846 (CH aliphatic), 2222 (C≡N), 1676 and 1654 (C=O), 1527–1512 (C=N), 1492–1473 (C=C); ^1^H NMR (400 MHz, DMSO‐*d*_*6*_) δ 4.34 (s, 2H, ‐CH_2_‐), 7.31 (t, 1H, thiophene H), 7.35 (s, 1H, Ar‐H), 7.45 (d, *J* = 7.9 Hz, 1H, Ar‐H), 7.68 (d, *J* = 6.7 Hz, 1H, Ar‐H), 8.05 (d, *J* = 4.8 Hz, 1H, thiophene H), 8.27 (d, *J* = 3.9 Hz, 1H, thiophene H), 10.10 (s, 1H, D_2_O exchangeable, ‐NH‐);^13^C NMR (100 MHz, DMSO‐*d*_*6*_) δ 35.44 (‐CH_2_‐), 88.72, 116.57 (C≡N), 124.75, 125.17, 127.18, 128.48, 129.85, 132.34, 132.41, 135.38, 137.10, 139.55, 159.18, 161.28, 165.39 (C=O amide), 166.03 (C=O amide); MS: m/z (%) 437.03 (M^+^+2, 67.60) 439.59 (M^+^+4, 24.41), 106.33 (100); Anal. Calcd. for C_17_H_10_Cl_2_N_4_O_2_S_2_ (435.31): C, 46.69; H, 2.30; N, 12.81; found: C, 46.65; H, 2.33; N, 12.94.

##### 2.1.1.12 *N*‐(4‐Chlorophenyl)‐2‐[(5‐Cyano‐6‐Oxo‐4‐(Thiophen‐2‐Yl)‐1,6‐Dihydropyrimidin‐2‐Yl)thio]Acetamide (3 l)

Orange powder (yield 75%), m.p. 235°C–240°C; IR (KBr) (cm^−1^): 3375–3325 (2NH), 3150–3039 (CH aromatic), 2927–2854 (CH aliphatic), 2222 (C≡N), 1693–1654 (C=O), 1597–1566 (C=N), 1527–1492 (C=C); ^1^H NMR (400 MHz, DMSO‐*d*_*6*_) δ 4.23 (s, 2H, ‐CH2‐), 7.28 (t, *J* = 4.4 Hz, 1H, thiophene H), 7.37 (d, *J* = 8.5 Hz, 2H. Ar‐H), 7.62 (d, *J* = 8.5 Hz, 2H, Ar‐H), 8.00 (d, *J* = 5.0 Hz, 1H, thiophene H), 8.24 (d, *J* = 3.9 Hz, 1H, thiophene H), 10.55 (s, 1H, ‐NH‐).^13^C NMR (100 MHz, DMSO‐*d*_*6*_) δ 35.88 (‐CH_2_‐), 88.71, 116.62, 121.27, 127.48, 129.17, 129.82, 132.32, 135.28, 138.31, 139.51, 159.17, 161.34, 165.39 (C=O amide), 165.63 (C=O amide); MS: m/z (%) 404.33 (M^+^+2,43.56), 402.53 (M^+^, 8.18), 70.42 (100); Anal. Calcd. for C_17_H_11_ClN_4_O_2_S_2_ (402.87): C, 50.68; H, 2.75; N, 13.91; found: C, 50.71; H, 2.70; N, 14.05.

##### 2.1.1.13 6‐Oxo‐2‐[(2‐Oxo‐2‐(*p*‐Tolyl)ethyl)thio]‐4‐(Pyridin‐3‐Yl)‐1,6‐Dihydropyrimidine‐5‐Carbonitrile (5a)

Orange powder (yield 78%), m.p. 240°C–242°C; IR (KBr) (cm^−1^): 3371–3336 (NH), 3074–3032 (CH aromatic), 2962–2920 (CH aliphatic), 2218 (C≡N), 1710 and 1678 (C=O), 1600.92–1585 (C=N), 1482–1469 (C=C); ^1^H NMR (400 MHz, DMSO‐*d*_*6*_) δ 2.40 (s, 3H, ‐CH_3_), 3.55 (s, 1H, ‐NH‐), 4.95 (s, 2H, ‐CH_2_‐), 7.34 (d, *J* = 8.3 Hz, 2H, Ar‐H), 7.65 (dd, *J* = 4.1, 8.1 Hz, 1H, pyridine H), 7.93 (d, *J* = 7.8 Hz, 2H, Ar‐H), 8.02 (d, *J* = 7.9 Hz, 1H, pyridine H), 8.68 (d, *J* = 4.9 Hz, 1H, pyridine H), 8.91 (s, 1H, pyridine H). ^13^C NMR (100 MHz, DMSO‐*d*_*6*_) δ 21.69 (‐CH_3_), 38.80 (‐CH_2_‐), 94.36, 115.96 (C≡N), 123.73, 128.85, 129.82, 131.36, 133.55, 136.55, 144.78, 149.26, 152.32, 161.17, 165.34, 166.63 (C=O amide), 192.70 (C=O ketone). MS: m/z (%) 362.37 (M^+^, 13.04), 339.41 (100); Anal. Calcd. for C_19_H_14_N_4_O_2_S (362.41): C, 62.97; H, 3.89; N, 15.46; found: C, 62.75; H, 4.06; N, 15.70.

##### 2.1.1.14 2‐[(2‐(4‐Bromophenyl)‐2‐Oxoethyl)thio]‐6‐Oxo‐4‐(Pyridin‐3‐Yl)‐1,6‐Dihydropyrimidine‐5‐Carbonitrile (5b)

Orange powder (yield 88%), m.p. 242°C–245°C; IR (KBr) (cm^−1^): 3451–3325 (NH), 3069–3066 (CH aromatic), 2997–2920 (CH aliphatic), 2202 (C≡N), 1693 and 1670 (C=O), 1595–1585 (C=N), 1543–1465 (C=C); ^1^H NMR (400 MHz, DMSO‐d6) δ 3.65 (s, 1H, ‐NH‐), 4.94 (s, 2H, ‐CH_2_‐), 7.39 (dd, *J* = 4.8, 7.8 Hz, 1H, pyridine H), 7.73 (d, *J* = 8.5 Hz, 2H, Ar‐H), 7.95 (d, *J* = 8.5 Hz, 2H, Ar‐H), 8.02 (d, *J* = 7.9 Hz, 1H, pyridine H), 8.68 (d, *J* = 3.2 Hz, 1H, pyridine H), 8.86 (s, 1H, pyridine H), ^13^C NMR (100 MHz, DMSO‐*d*_*6*_) δ 38.66 (‐CH_2_‐), 94.51, 115.91 (C≡N), 123.67, 128.36, 130.70, 131.33, 132.32, 135.15, 136.47, 149.28, 152.31, 161.16, 165.40, 166.47 (C=O amide), 192.72 (C=O ketone); MS: m/z (%) 429.14 (M^+^+2, 13.84) 427.37 (M^+^, 14.89), 74.87 (100); Anal. Calcd. for C_18_H_11_BrN_4_O_2_S (427.28): C, 50.60; H, 2.60; N, 13.11; found: C, 50.82; H, 2.71; N, 13.29.

##### 2.1.1.15 6‐Oxo‐2‐[(2‐Oxo‐2‐(*p*‐Tolyl)ethyl)thio]‐4‐(Thiophen‐2‐Yl)‐1,6‐Dihydropyrimidine‐5‐Carbonitrile (5c)

Orange powder (yield 79%), m.p. 230°C–232°C; IR (KBr) (cm^−1^): 3361–3298 (NH), 3105–3070 (CH aromatic), 2966–2927 (CH aliphatic), 2222 (C≡N), 1693 and 1654 (C=O), 1605–1585 (C=N), 1543–1469 (C=C); ^1^H NMR (400 MHz, DMSO‐*d*_*6*_) δ 2.43 (s, 3H, ‐CH_3_), 4.97 (s, 2H, ‐CH_2_‐), 7.23 (t, *J* = 4.5 Hz, 1H, thiophene H), 7.40 (d, *J* = 7.9 Hz, 2H, Ar‐H), 7.91 (d, *J* = 5.0 Hz, 1H, thiophene H), 7.99 (d, *J* = 7.9 Hz, 2H, Ar‐H), 8.13 (d, *J* = 4.0 Hz, 1H, thiophene H), 13.85 (s,1H, D_2_O exchangeable, ‐NH‐); ^13^C NMR (100 MHz, DMSO‐*d*_*6*_) δ 21.74 (‐CH_3_), 39.28 (‐CH_2_‐), 88.44, 116.66 (C≡N), 128.97, 129.78, 129.89, 132.02, 133.43, 135.20, 139.56, 144.85, 158.96, 161.43, 165.55 (C=O amide), 191.94 (C=O ketone); MS: m/z (%) 367.43 (M^+^, 20.80), 97.58 (100) Anal. Calcd. for C_18_H_13_N_3_O_2_S_2_ (367.44): C, 58.84; H, 3.57; N, 11.44; found: C, 59.06; H, 3.68; N, 11.71.

##### 2.1.1.16 2‐[(2‐(4‐Bromophenyl)‐2‐Oxoethyl)thio]‐6‐Oxo‐4‐(Thiophen‐2‐Yl)‐1,6‐Dihydropyrimidine‐5‐Carbonitrile (5d)

Orange powder (yield 89%), m.p. 237°C–240°C; IR (KBr) (cm^−1^): 3383–3326 (NH), 3101–3035 (CH aromatic), 2958–2912 (CH aliphatic), 2222 (C≡N), 1701 and 1670 (C=O), 1585–1566 (C=N), 1531–1462 (C=C); ^1^H NMR (400 MHz, DMSO‐*d*_*6*_) δ 4.98 (s, 2H, ‐CH_2_‐), 7.23 (t, 1H, thiophene H), 7.87 (d, *J* = 30.7 Hz, 3H, thiophene H, Ar‐H), 8.08 (d, *J* = 40.0 Hz, 3H, thiophene H, Ar‐H), 13.85 (s, 1H, ‐NH‐); ^13^C NMR (100 MHz, DMSO‐*d*_*6*_) δ 31.16 (‐CH_2_‐), 88.56, 116.52 (C≡N), 128.51, 129.83, 130.83, 132.10, 132.44, 134.88, 135.25, 139.40, 158.93, 161.19, 165.23 (C=O amide), 191.70 (C=O ketone); MS: m/z (%) 434.59 (M^+^+2, 24.21), 432.68 (M^+^, 100); Anal. Calcd. for C_17_H_10_BrN_3_O_2_S_2_ (432.31): C, 47.23; H, 2.33; N, 9.72; found: C, 47.31; H, 2.36; N, 9.81.

##### 2.1.1.17 2‐[(5‐Cyano‐6‐Oxo‐4‐(Pyridin‐3‐Yl)‐1,6‐Dihydropyrimidin‐2‐Yl)thio]‐*N*‐[4‐(*p*‐Tolyl)thiazol‐2‐Yl]Acetamide (7a)

Orange powder (yield 79%), m.p. 250°C–253°C; IR (KBr) (cm^−1^): 3336–3232 (2NH), 3066–3035 (CH aromatic), 2916–2850 (CH aliphatic), 2222 (C≡N), 1689 and 1638 (C=O), 1585–1573 (C=N), 1565–1535 (C=C); ^1^H NMR (400 MHz, DMSO‐*d*_*6*_) δ 2.34 (s, 3H, ‐CH_3_), 4.39 (s, 2H, ‐CH_2_‐), 5.07 (s, 1H, D_2_O exchangeable, ‐NH‐), 7.26 (d, *J* = 7.9 Hz,2H, Ar‐H), 7.53 (s, 1H, ‐CH‐ thiazole H), 7.63 (dd, *J* = 5.1, 8.0 Hz, 1H, pyridine H), 7.79 (d, *J* = 7.8 Hz, 2H, Ar‐H), 8.49 (d, *J* = 8.1 Hz, 1H, pyridine H), 8.76 (d, *J* = 3.7 Hz, 1H, pyridine H), 9.22 (s, 1H, pyridine H), 12.65 (s, 1H, D_2_O exchangeable, ‐NH‐); ^13^C NMR (100 MHz, DMSO‐*d*_*6*_) δ 21.31 (‐CH_3_‐), 34.87 (‐CH_2_‐), 95.32, 107.85, 115.63 (C≡N), 125.41, 126.11, 129.81, 131.95, 132.81, 137.30, 137.64, 140.67, 146.18, 149.46, 157.85, 160.94, 163.88, 166.43 (C=O amide), 166.89 (C=O amide); MS: m/z (%) 460.63 (M^+^, 23.71), 383.30 (100) Anal. Calcd. For C_21_H_16_N_6_OS_2_ (460.53): C, 57.38; H, 3.50; N, 18.25; found: C, 57.57; H, 3.68; N, 18.26.

##### 2.1.1.18 *N*‐(4‐(4‐*B*romophenyl)thiazol‐2‐Yl)‐2‐((5‐Cyano‐6‐Oxo‐4‐(Thiophen‐2‐Yl)‐1,6‐Dihydropyrimidin‐2‐Yl)thio)acetamide (7b)

Pale yellow powder (yield 75%), m.p. 248°C–250°C; IR (KBr) (cm‐1): 3290–3167 (2NH), 3105–3070 (CH aromatic), 2916–2850 (CH aliphatic), 2222 (C≡N), 1705 and 1654 (C=O), 1543–1532 (C=N), 1510–1473 (C=C); ^1^H NMR (400 MHz, DMSO‐*d*_*6*_) δ 4.38 (s, 2H, ‐CH_2_‐), 7.24 (t, *J* = 4.49 Hz, 1H, thiophene H), 7.66 (d, *J* = 8.2 Hz, 2H, Ar‐H), 7.70 (s, 1H, ‐CH‐ thiazole H), 7.88 (d, *J* = 8.1 Hz, 2H, Ar‐H), 7.92 (d, *J* = 5.0 Hz, 1H, thiophene H), 8.23 (d, *J* = 3.9 Hz, 1H, thiophene H), 12.79 (s, 1H, D_2_O exchangeable, ‐NH‐), 13.83 (s, 1H, D_2_O exchangeable, ‐NH‐), ^13^C NMR (100 MHz, DMSO‐*d*_*6*_) δ 34.78 (‐CH_2_), 88.81, 109.56, 116.60 (C≡N), 121.39, 128.18, 129.86, 132.20, 132.30, 133.89, 135.14, 139.45, 148.22, 158.58, 159.15, 161.34165.33 (C=O amide), 166.05 (C=O amide); MS: m/z (%) 530.93 (M+, 23.71), 201.04 (100); Anal. Calcd. for C_19_H_14_BrN_5_OS_3_ (530.43): C, 45.29; H, 2.28; N, 13.20; found: C, 45.43; H, 2.23; N, 13.28.

### 2.2 Biological Evaluation

#### 2.2.1 The Agar Well Diffusion Method

The antimicrobial activity was evaluated using the well diffusion method as previously described (Magaldi et al. 2004; Valgas et al. 2007). For antibacterial assessment, *Staphylococcus aureus* ATCC 29213 and *Bacillus subtilis* ATCC 6633 were employed as Gram‐positive strains, while *Pseudomonas aeruginosa* ATCC 41501 served as a Gram‐negative strain. Bacterial suspensions were adjusted to a 0.5 McFarland standard, corresponding to approximately 1 × 10^6^–1.5 × 10^6^ CFU/mL, to ensure consistent inoculum density. Tested Compounds were dissolved in DMSO, with final concentration of 5% (v/v) in this assay. A 20 μL aliquot of the test compound at the desired concentration was introduced into each well of Mueller–Hinton Agar (MHA) plates, selected for its suitability in supporting a broad range of bacterial growth and its standardized application in susceptibility testing. Pure 5% DMSO was used as negative control, and amoxicillin served as the positive control. Plates were incubated at 37°C for 24 h. Antimicrobial activity was determined by measuring the diameter of the clear inhibition zones formed around the wells, reflecting the diffusion of the compound into the medium and its inhibitory effect on microbial growth. All assays were performed in triplicates, and the results are expressed as mean.

#### 2.2.2 The Agar Disk Diffusion for *β*‐Lactamase Resistant Strains

The In vitro *β*‐Lactamase inhibition assay was performed using the agar disk diffusion method, following a previously established procedure. The plates were inoculated with a bacteria strain with known *β*‐lactamase production properties such as *Acinetobacter baumannii* ATCC 19606, *Bacillus subtilis* ATCC 6633 (Uri et al. 1978), and *Staphylococcus aureus* ATCC 29213 (Zygmunt et al. 1992). The inhibition zone is then measured and compared with the negative control which is a disk containing either ampicillin or penicillin alone to confirm the baseline resistance of the strains. To evaluate potential *β*‐Lactamase inhibition, test compounds were combined with these antibiotics to observe the restoration of inhibitory activity. Additionally, a vehicle control consisting of disks impregnated with 5% DMSO was added to verify that the solvent lacked any intrinsic antibacterial activity. All assays were performed in triplicates, and the results are expressed as mean.

#### 2.2.3 In Vitro *β*‐Lactamase Enzyme Activity Assay

The *β*‐lactamase inhibitor screening assay was performed using kit ab197009 Beta Lactamase Inhibitor Screening Assay Kit (Colorimetric) introducing nitrocefin as the chromogenic substrate. Test compounds were dissolved in appropriate solvents and diluted with assay buffer to 5X the desired concentration. In a 96‐well plate, A reaction mixture containing 48 μL assay buffer and 2 μL *β*‐lactamase was added to each well, followed by incubation for 10 min at 25°C. Subsequently, 30 μL of substrate mixture (29 μL assay buffer +1 μL nitrocefin) was added, mixed, and the absorbance was recorded immediately at 490 nm (T₁) and at 3 min intervals for 30 min at room temperature in the dark. Reaction rates (slopes) were calculated as:

(Slope) $$\frac{A2-A1}{T2-T1}\times D$$

where A1 and A2 are absorbance values at times T1 and T2, respectively, and D is the dilution factor.

The % relative inhibition was determined using the formula:

$$\%\text{relative inhibition}=\frac{\;\text{SlopeEC}-\text{SlopeS}}{\text{SlopeEC}}\times 100$$

where *Slope*EC is the enzyme control and *Slope*S is the sample.

#### 2.2.4 MIC‐Modifying Effects of the Promising Target Compounds

The broth microdilution method was used in 96‐well plates to assess the Minimum Inhibitory Concentration (MIC) of ampicillin and the MIC‐modifying effects of the promising target compounds with possible beta‐lactamase inhibitory action. The experiment was conducted using a clinical strain of *Staphylococcus aureus* ATCC 29213 with reported *β*‐lactamase activity (Zygmunt et al. 1992). The experiment's initial step was to determine the baseline MIC of ampicillin alone using a twofold serial dilution in Mueller‐Hinton broth to evaluate the combined effects of ampicillin and the target compounds (diluted in 5% DMSO). Both positive (Broth + Bacteria) and negative (Broth only) growth controls were used during this phase. A sub‐MIC ampicillin concentration was chosen for the second phase based on the findings of the first phase. All wells received a fixed, sub‐MIC concentration of ampicillin, and each drug was twofold serially diluted on the plate. Controls for the compounds alone were added in this phase to verify that it lacked inhibitory activity. Additionally, DMSO 5% was tested to confirm the absence of any intrinsic antibacterial effect. 5 μL of the standardized bacterial inoculum (adjusted to 0.5 McFarland) was introduced to each well after all dilutions had been prepared. The plates were incubated at 37°C for 24 h. After that, the MIC values were noted by visual inspection of turbidity. The experiment was conducted in triplicate (Kadeřábková et al. 2024).

### 2.3 In Silico Study

#### 2.3.1 Molecular Docking Study

Target compounds were sketched using MarvinSketch and energy‐minimized in Avogadro employing the MMFF94s force field. The crystal structure of CTX‐M‐9 class A *β*‐lactamase (PDB ID: 4DDS) was retrieved from the RCSB Protein Data Bank (Burley et al. 2019; Nichols et al. 2012). and prepared using the *Make Receptor* function of the OEDOCKING 3.4.0.2 module generating a grid box dimension to 22.70 Å in length, 17.17 Å width, and 15.27 Å in height, providing a workspace of approximately 5953 Å for the HYBRID docking algorithm (OpenEye) (OpenEye, Cadence Molecular Sciences Inc, n.d.‐a).

Ligand conformers were generated with OMEGA 3.1.2.2 (OpenEye) and docked using the HYBRID function of OEDOCKING (Hawkins et al. 2010; OpenEye, Cadence Molecular Sciences Inc, n.d.‐b). The DockRMSD metric was employed to calculate the (RMSD) of the docked compounds (Bell and Zhang 2019). The validated docking procedure was then employed to analyze the ligand‐target interactions in the active site for the most potent synthesized compounds **3d**, **3i**, and **7b** to anticipate their binding and justify their activity. Protein–ligand complexes were visualized in 3D using VIDA (OpenEye, Cadence Molecular Sciences Inc, n.d.‐c) and in 2D using Discovery Studio Visualizer (BIOVIA Dassault Systèmes 2021).

#### 2.3.2 Toxicity Prediction

Toxicity simulations are performed using pkCSM web tool (https://biosig.lab.uq.edu.au/pkcsm) by entering SMILES codes of the target structures and evaluating results based on the ranges and referenced offered by the web tool (Pires et al. 2015).

## 3 Results and Discussion

### 3.1 Chemistry

**SCHEME 1:**
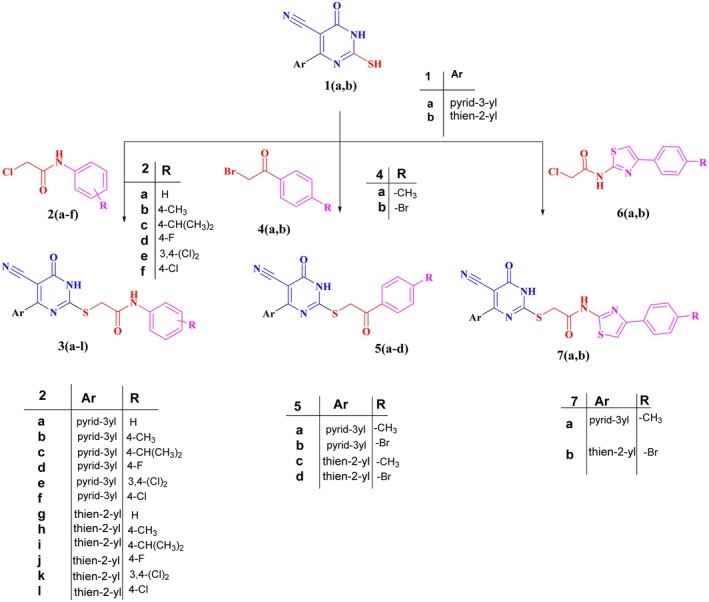
General steps for preparation of target compounds. **Reagents and Reaction Conditions:** Triethylamine, acetone, potassium carbonate, potassium iodide, reflux (20–24) h. Alkylation of DHPMs **1 (a,b)** with the appropriate *α*‐halo carbonyl compounds **2 (a‐f)**, **4 (a,b)**, and **6 (a,b)** gave the target candidates **3 (a‐l)**, **5 (a‐d)**, and **7 (a,b)** respectively. For compounds **3 (a‐l)**, IR spectra showed significant stretching bands at 3420–3230 cm^−1^ assigned to 2NH amidic groups, 2220–2218 cm^−1^ related to C≡N group, Two amidic C=O groups stretching bands appear at 1693–1678 cm^−1^ and 1658–1647 cm^−1^.

Synthesis of the target compounds has been achieved through the steps described in Scheme 1. Nucleophilic substitution of the thiol group in the core starting compound **1** results in the formation of the target compounds.

**FIGURE 4:**
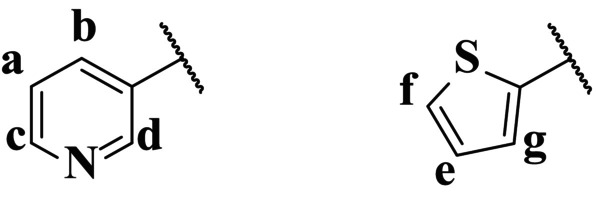
Pyrid‐3‐yl and thien‐2‐yl protons.

^1^H NMR spectra for compounds **3 (a‐*l*)** showed significant singlet peak at *δ* 3.8–4.3 ppm related to (‐CH_2_ ‐). Two singlet peaks of the (‐NH) were detected; one in the range of (*δ* 4.4–7.8) ppm, while the second one was in the range of (*δ* 10.41–13.67) ppm and both were disappeared by deuteration. Compounds **3 (a‐f)** showed a distinctive pattern related to the pyridyl protons where the four aromatic protons appear as doublet of doublet (a) doublet (b), doublet (c) and singlet (d) in the range of (*δ* 7.15–9.8) ppm (Figure 4). Compounds **3 (g‐l)** showed a distinctive pattern related to the thienyl protons where the three aromatic protons appear as triplet (e), doublet (f) and doublet (g) in the range of *δ* (7.16–8.3) ppm (Figure 4).

Moreover, compounds **3a** and **3g** showed two triplet peaks (*δ* 7.1 ppm and 7.24 ppm, respectively) and a doublet peak (*δ* 7.501 ppm) corresponding to monosubstituted phenyl ring protons. Compounds **3b**, **3c, 3h**, and **3i** showed the aromatic pattern of two doublets characteristic of the *para* substitution at (δ 7.10 and 7.45 ppm, respectively). Compounds **3b** and **3h** showed a singlet peak at (*δ* 2.24–2.56) ppm for (‐CH_3_). Compounds **3c** and **3i** showed doublet and multiplet peaks (1.21 and 2.8 ppm, respectively) which are consistent with isopropyl protons. **3d** and **3j** showed the two distinctive patterns of doublet and triplet (*δ* 7.14 and 7.49 ppm, respectively) which resulted from the effect of the fluorine atom on the neighboring protons. **3e** and **3k** showed three peaks (*δ* 7.25–7.67) ppm corresponding to the aromatic protons. Compounds **3f** and **3l** showed two doublets in the range of (*δ* 7.33–7.62) ppm for the aromatic protons.

For compounds **5 (a‐d)**, IR spectra showed significant stretching bands at 3451–3298 cm^−1^ assigned to the NH amidic group, 2222–2202 cm^−1^ related to the CN group, and the C=O group stretching band appears at 1693 cm^−1^, and amidic C=O 1670 cm^−1^.

^1^H NMR charts of the mentioned compounds showed only one singlet peak of NH. **5 (a,b)** showed an NH peak in the range of (*δ* 3.61–3.62) ppm. **5 (c,d)** showed an NH peak in the range of (*δ* 13.85–13.87) ppm. **5 (a‐d)** showed a significant singlet peak related to (‐CH_2_‐) in the range of (*δ* 4.94–4.97 ppm).

For compounds **7 (a‐b)**, IR spectra showed two bands of the 2 NH amidic group stretching vibrations at 3390–3160 cm^−1^, stretching band of the CN group at 2220 cm^−1^, and two amidic C=O groups' stretching bands appear at 1689–1654 cm^−1^.

^1^H NMR showed in both compounds a significant singlet peak of (‐CH‐) thiazole at range of (*δ* 7.53–7.70) ppm. Moreover, the singlet peak of (‐CH_2_‐) aliphatic protons appeared in the range of (*δ* 4.38–4.39) ppm. **7a** shows two NH exchangeable protons with D_2_O deuterated that were detected at *δ* 5.07 and 12.65 ppm. On the other hand, **7b** shows two NH exchangeable protons with D_2_O that were reported at (*δ* 12.79 and 13.83) ppm. **7 (a,b)** reported the distinctive aromatic pattern of two doublet signals in the range of (*δ* 7.26–7.88) ppm. **7a** CH_3_ protons were reported at (*δ* 2.34) ppm. ^13^C NMR and mass spectra of the target compounds were consistent with the expected pattern.

### 3.2 Biological Activity

#### 3.2.1 The Agar Well Diffusion Method

**FIGURE 5:**
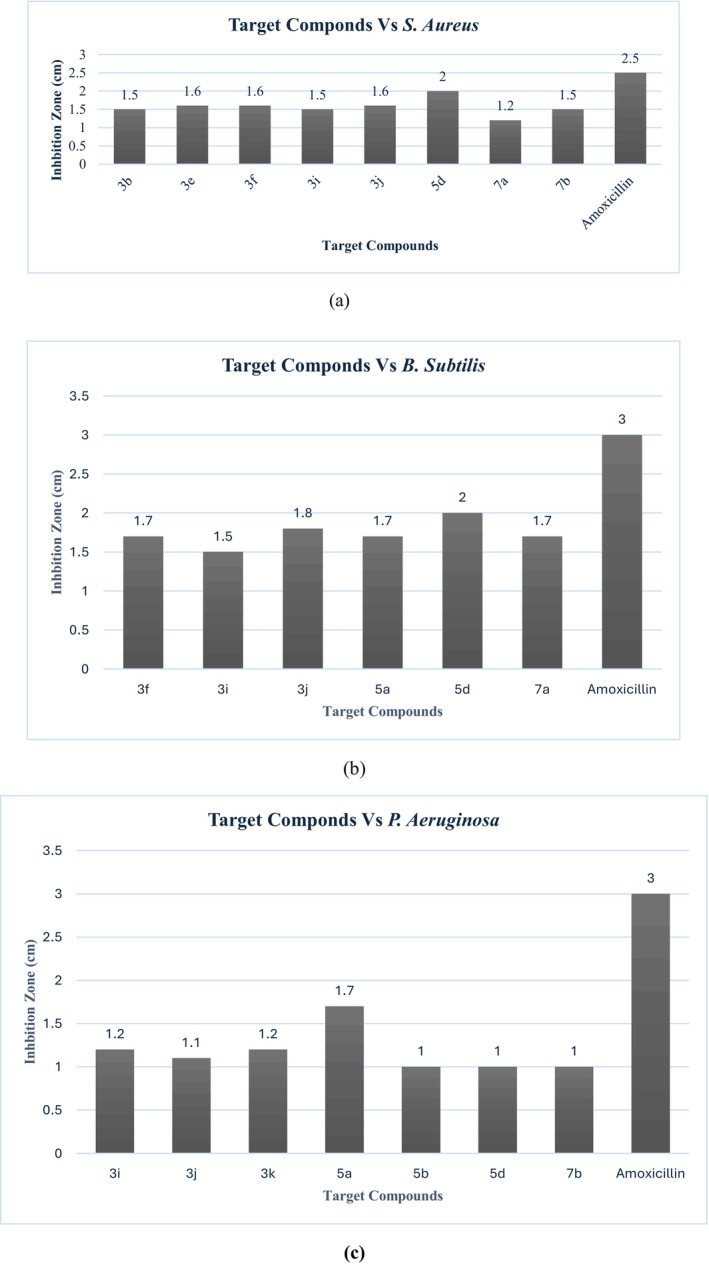
Inhibitions zones (cm) of the target compounds vs. amoxicillin against *S. aureus* (a), *B. subtilis* (b), and *P. aeruginosa* (c).

All the newly synthesized compounds were evaluated for their antimicrobial activity against specific bacterial species *Staphylococcus aureus* ATCC 29213, *Bacillus subtilis* ATCC 6633 as a gram‐positive species, whereas *Pseudomonas aeruginosa* ATCC 41501 as a gram‐negative species using amoxicillin as a reference drug, showing potential effectiveness. DMSO 5% was tested as vehicle control and showed no zone of inhibition against the tested strains. Compounds **3b**, **3e**, **3f**, and **3j** demonstrated moderate inhibition zones against *S. aureus* , while **3f**, **4a**, and **5a** showed similar results against *B. subtilis* . **5a** had the largest inhibitory zone against *P. aeruginosa* . Additionally, compounds **3i**, **3j**, and **5d** exhibited comparable inhibitory zones against the selected bacteria. The results raised the probability of the ability of those compounds to have a potentiation effect not a direct antimicrobial activity (Table 1; Figure 5; Figure S37). Analysis of the previous data indicated that the most effective compounds, **3i**, **3j**, and **5d** against the selected bacterial species were substituted at position 4‐ with 2‐thienyl.

**TABLE 1:** Inhibition zones (cm) of target compounds versus amoxicillin.

Compound	*S. aureus*	*B. subtilis*	*P. aeruginosa*
	Mean ± SD	Mean ± SD	Mean ± SD
**3a**	—	—	—
**3b**	1.5 ± 0.1	—	—
**3c**	—	—	—
**3d**	—	—	—
**3e**	1.6 ± 0.1	—	—
**3f**	1.6 ± 0.1	1.7 ± 0.08	—
**3g**	—	—	—
**3h**	—	—	—
**3i**	1.5 ± 0.06	1.5 ± 0.1	1.2 ± 0.06
**3j**	1.6 ± 0.06	1.8 ± 0.05	1.1 ± 0.06
**3k**	—	—	1.2 ± 0.06
**3l**	—	—	—
**5a**	—	1.7 ± 0.05	1.7 ± 0.06
**5b**	—	—	1 ± 0.06
**5c**	—	—	—
**5d**	2 ± 0.06	2 ± 0.06	1 ± 0.15
**7a**	1.2 ± 0.06	1.7 ± 0.01	—
**7b**	1.5 ± 0.1	—	1 ± 0.06
Amoxicillin	2.5 ± 0.06	3 ± 0.15	3 ± 0.06
DMSO 5%	—	—	—

#### 3.2.2 The Agar Disk Diffusion for *β*‐Lactamase Resistant Strains

The method aimed to verify whether the synthesized compounds could enhance the effectiveness of *β*‐lactamase susceptible antibiotics, such as penicillin and ampicillin, against *β*‐lactamase resistant strains of *S. aureus* ATCC 29213, *B. subtilis* ATCC 6633, and *A. baumannii* ATCC 19606. DMSO 5% was tested as vehicle control and showed no zone of inhibition against the tested strains.

**FIGURE 6:**
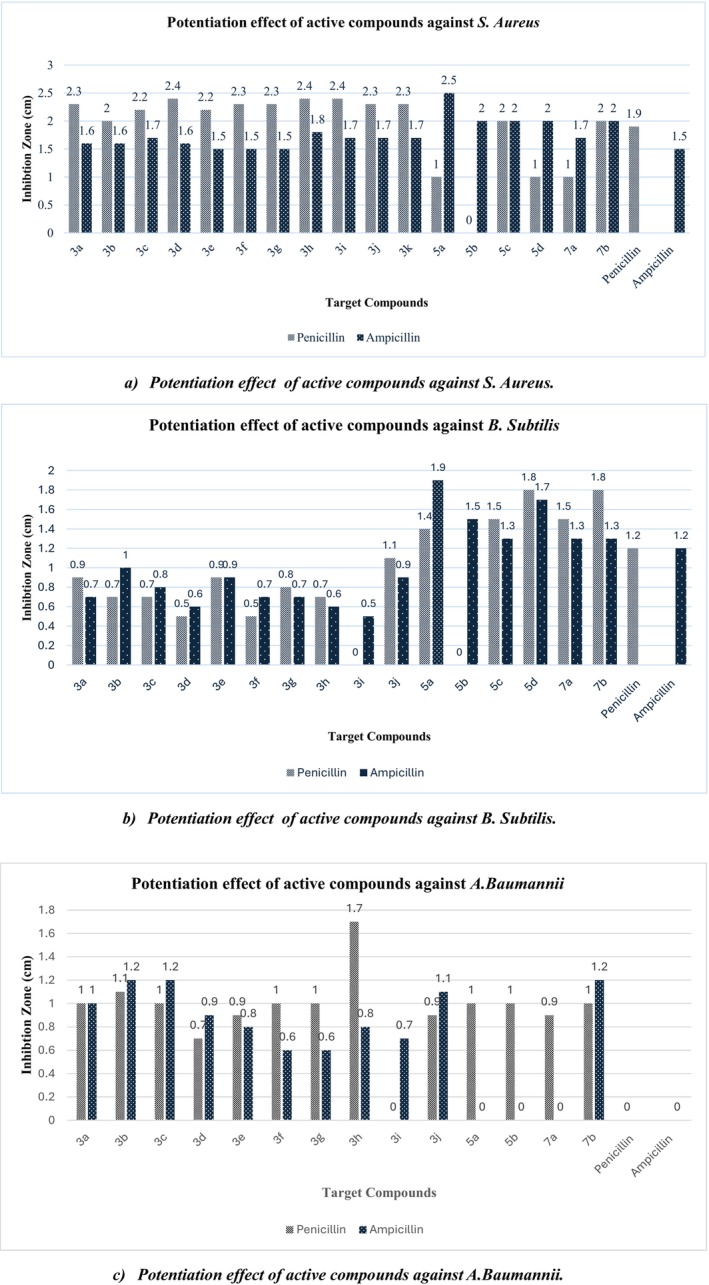
Charts of inhibition zones resulting from the potentiation effect of target compounds with antibiotics against (a) *S. aureus* , (b) *B. subtilis* , and (c) *A. baumannii* .

The results presented in Table 2 and Figure 6 indicate that nearly all the target compounds increased the inhibitory effectiveness of antibiotics against the resistant strains of *S. aureus* , exceeding the effects of penicillin and ampicillin used alone. Specifically, target compounds **3d**, **3h**, and **3i** demonstrated the largest inhibition zones, measuring 2.4 cm with penicillin and 1.9 cm with ampicillin, compared to 1.9 cm and 1.5 cm for penicillin and ampicillin individually (Figure S38).

**TABLE 2:** The inhibition zones (cm) resulted in the potentiation effect of the target compounds with antibiotics.

Compound	*S. aureus*		*B. subtilis*		*A. baumannii*	
	Penicillin	Ampicillin	Penicillin	Ampicillin	Penicillin	Ampicillin
**3a**	2.3 ± 0.06	1.6 ± 0.06	0.9 ± 0.06	0.7 ± 0.06	1 ± 0.06	1 ± 0.06
**3b**	2 ± 0.1	1.6 ± 0.08	0.7 ± 0.06	1 ± 0.06	1.1 ± 0.06	1.2 ± 0.06
**3c**	2.2 ± 0.03	1.7 ± 0.12	0.7 ± 0.06	0.8 ± 0.06	1 ± 0.06	1.2 ± 0.06
**3d**	2.4 ± 0.1	1.6 ± 0.06	0.5 ± 0.06	0.6 ± 0.06	0.7 ± 0.06	0.9 ± 0.06
**3e**	2.2 ± 0.06	1.5 ± 0.06	0.9 ± 0.06	0.9 ± 0.06	0.9 ± 0.1	0.8 ± 0.1
**3f**	2.3 ± 0.06	1.5 ± 0.05	0.5 ± 0.06	0.7 ± 0.06	1 ± 0.06	0.6 ± 0.06
**3g**	2.3 ± 0.08	1.5 ± 0.13	0.8 ± 0.1	0.7 ± 0.1	1 ± 0.06	0.6 ± 0.06
**3h**	2.4 ± 0.06	1.8 ± 0.06	0.7 ± 0.06	0.6 ± 0.06	1.7 ± 0.06	0.8 ± 0.06
**3i**	2.4 ± 0.06	1.7 ± 0.06	—	0.5 ± 0.06	—	0.7 ± 0.06
**3j**	2.3 ± 0.06	1.7 ± 0.06	1.1 ± 0.12	0.9 ± 0.1	0.9 ± 0.06	1.1 ± 0.12
**3k**	2.3 ± 0.1	1.7 ± 0.03	—	—	—	—
**3l**	—	—	—	—	—	—
**5a**	1 ± 0.06	2.5 ± 0.06	1.4 ± 0.06	1.9 ± 0.1	1 ± 0.06	—
**5b**	—	2 ± 0.06	—	1.5 ± 0.06	1 ± 0.06	—
**5c**	2 ± 0.05	2 ± 0.06	1.5 ± 0.06	1.3 ± 0.06	—	—
**5d**	1 ± 0.06	2 ± 0.06	1.8 ± 0.06	1.7 ± 0.06	—	—
**7a**	1 ± 0.06	1.7 ± 0.03	1.5 ± 0.06	1.3 ± 0.05	0.9 ± 0.15	—
**7b**	2 ± 0.06	2 ± 0.06	1.8 ± 0.06	1.3 ± 0.06	1 ± 0.06	1.2 ± 0.15
Antibiotic alone	1.9 ± 0.1	—	1.2 ± 0.06	—	0	—
Antibiotic alone	—	1.5 ± 0.06	—	1.2 ± 0.06	—	0
DMSO 5%	—	—	—	—	—	—

On the other hand, compounds **5a‐d** and **7a,b** were the target compounds that enhanced the inhibition zone against *B. subtilis* when combined with penicillin and ampicillin, compared to their effects when used alone. Finally, the gram‐negative *A. baumannii* showed no inhibition zone with the reference antibiotics, but most target compounds increased the inhibition zone. Compound **3h** was the most active, exhibiting a 1.7 cm inhibition zone with penicillin.

The most potentiation effect was noted in compounds with electron‐withdrawing substituents, including **3d**, **3e**, **3f**, **3j**, **3k**, and **7b**.

#### 3.2.3 *β*‐Lactamase Enzyme Activity

**FIGURE 7:**
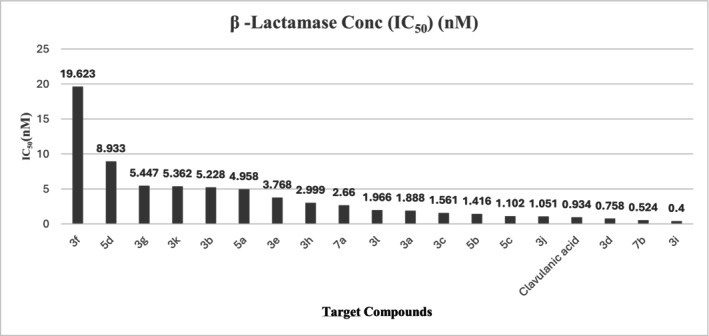
Chart of IC_50_ values from *β*‐Lactamase enzyme assay.

As noted in the previous experiment, the potentiation effect of the target compounds with antibiotics has been confirmed, likely by inhibiting *β*‐lactamase enzymes. The target compounds were subjected to in vitro enzymatic assay against *β*‐lactamase enzyme to assess their inhibitory activities against clavulanic acid as the reference drug. The synthesized compounds demonstrated promising IC_50_ values in comparison to reference *β*‐lactamase inhibitor, clavulanic acid (Table 3; Figure 7). Compound **3i** had the lowest IC_50_ at 0.400 nM, whereas clavulanic acid had an IC_50_ of 0.934 nM. Additionally, compounds **3d** and **7b** exhibited lower IC_50_ values of 0.758 and 0.524 nM, respectively.

**TABLE 3:** IC_50_ values of the tested compounds and clavulanic acid in in vitro *β*‐lactamase enzyme assay.

Compound	*β*‐lactamase conc (IC_50_) (nM)
**3a**	1.888 ± 0.069
**3b**	5.228 ± 0.191
**3c**	1.561 ± 0.057
**3d**	0.758 ± 0.029
**3e**	3.768 ± 0.139
**3f**	19.623 ± 0.719
**3g**	5.447 ± 0.201
**3h**	2.999 ± 0.110
**3i**	0.400 ± 0.015
**3j**	1.051 ± 0.039
**3k**	5.362 ± 0.198
**3l**	1.966 ± 0.072
**5a**	4.958 ± 0.080
**5b**	1.416 ± 0.042
**5c**	1.102 ± 0.038
**5d**	8.933 ± 0.671
**7a**	2.660 ± 0.413
**7b**	0.524 ± 0.040
Clavulanic acid	0.934 ± 0.035

The efficacy of compounds **3f**, **3k**, and **3e** in inhibiting the *β*‐lactamase enzyme was found to be inadequate, indicating that chlorinated compounds have limited potential as inhibitors. Among the fluorinated derivatives, **3i** displayed the highest inhibitory activity, while **3j** showed promising results compared to clavulanic acid. These findings suggest that activity is influenced not only by electron‐withdrawing substituents but also by the size of the atoms bearing these substituents. The methyl group, acting as an electron‐donating substituent, proved ineffective in compounds **3b** and **3h**, whereas the isopropyl group demonstrated greater effectiveness with lower IC_50_ values. In the absence of substitution, derivatives **3a** and **3g** also showed lower IC_50_ values.

Furthermore, compound **7b** demonstrated a significant IC_50_ value compared to clavulanic acid, while **7a** showed lower IC_50_ values, indicating that electron‐withdrawing substituents are more effective as inhibitors (Table S1; Figure S39).

#### 3.2.4 MIC‐Modifying Effects of the Promising Target Compounds

As the previous results indicated, there is a potentiation effect of **3d**, **3i**, and **7b** with different antibiotics. MIC study was conducted using ampicillin as a reference against the clinical strain of *S. aureus* with reported *β*‐lactamase activity. Baseline MIC of ampicillin was determined to be 0.075 mg/mL. The promising target compounds were then tested for their ability to inhibit bacterial growth in the presence of a fixed sub‐MIC concentration of ampicillin. The results showed that compound **3d** and **3i** demonstrated inhibition at 2.5 mg/mL, and compound **7b** showed inhibition at 0.625 mg/mL (Table 4).

**TABLE 4:** MIC concentration of the promising target compounds a fixed sub‐MIC concentration of ampicillin.

Compound	3d	3i	7b	Solvent control (5% DMSO)	Positive control	Negative control
					Bacterial broth	Bacterial broth
Inhibitory concentration	2.5 mg/mL	2.5 mg/mL	0.625 mg/mL	No inhibitory effect on all wells	Visible bacterial growth on all wells	No bacterial growth

As shown in (Table 4) the solvent control DMSO 5% showed no inhibitory activity, confirming that the antibacterial activity was not due to solvent. Furthermore, the positive control showed confirmed the viability of the bacterial strain in the broth medium.

### 3.3 In Silico Studies

#### 3.3.1 Molecular Docking Study

The identification of novel noncovalent inhibitors of class A *β‐*lactamases presents an approach in maintaining the efficacy of *β*‐lactam antibiotics. The derivatives **3d, 3i**, and **7b** in in vitro *β*‐lactamase assay were selected for the docking study. The Beta‐lactamase inhibitory activity was evaluated using a commercial Inhibitor Screening Assay Kit (Abcam, ab197009) according to the manufacturer's manual. The assay utilized an Escherichia coli strain expressing the enzyme, consistent with the structural model of the CTX‐M‐9 (PDB ID: 4DDS).

**FIGURE 8:**
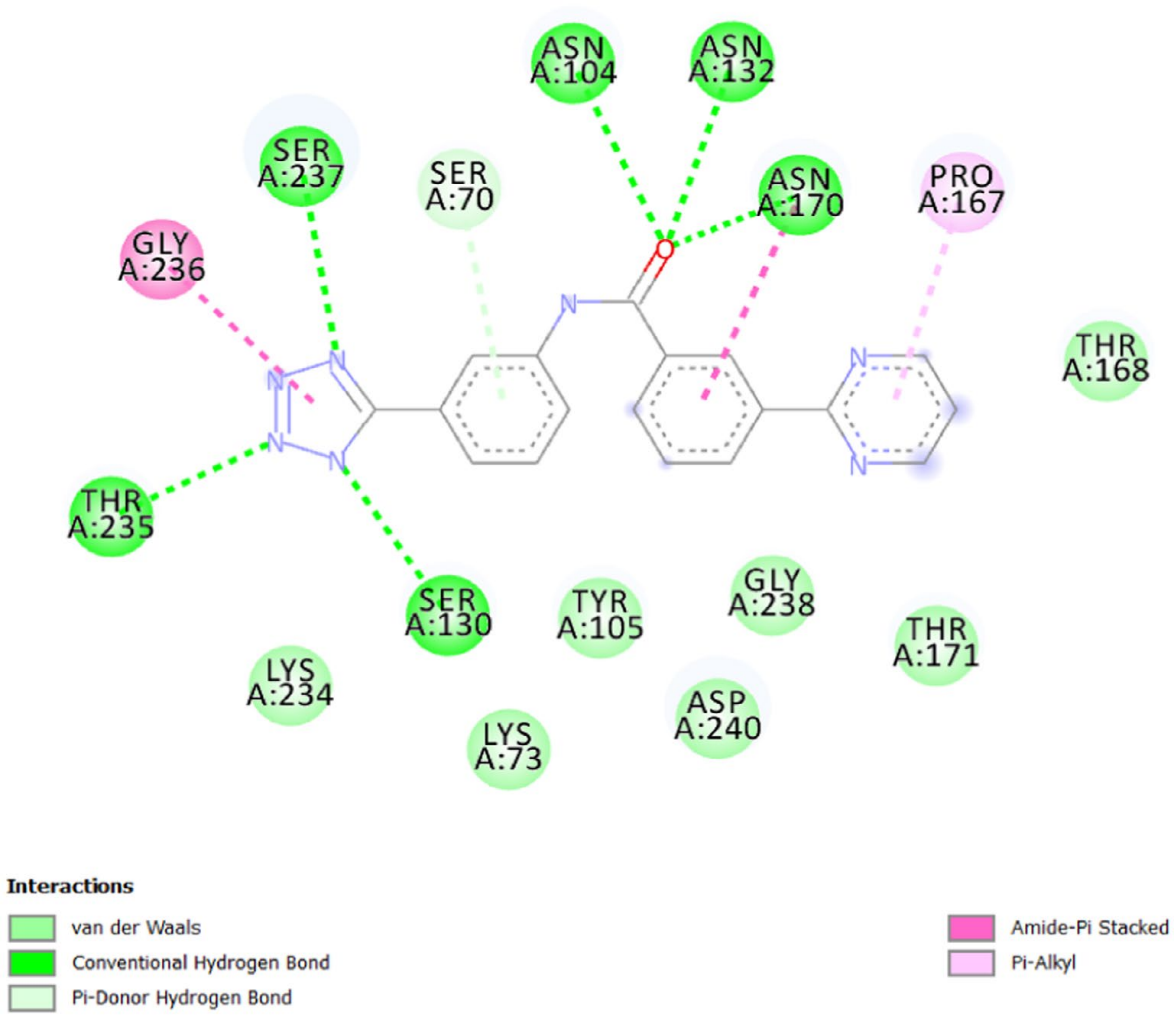
2D binding interaction between reference ligand and *β*‐lactamase enzyme binding site.

A literature review highlighted the significance of hydrogen bonds formed by various *β*‐lactamase inhibitors with key residues in the protein‐binding site, including Ser70, Asn132, Asn104, Thr235, Ser237, Asp240, and Ser130, as well as hydrophobic interactions with Pro167. These binding hotspots are essential considerations for designing effective inhibitors against (PDB:4DDS) as driven from the In vitro assay kit (Chen et al. 2005; Nichols et al. 2012; Figure 8).

**FIGURE 9:**
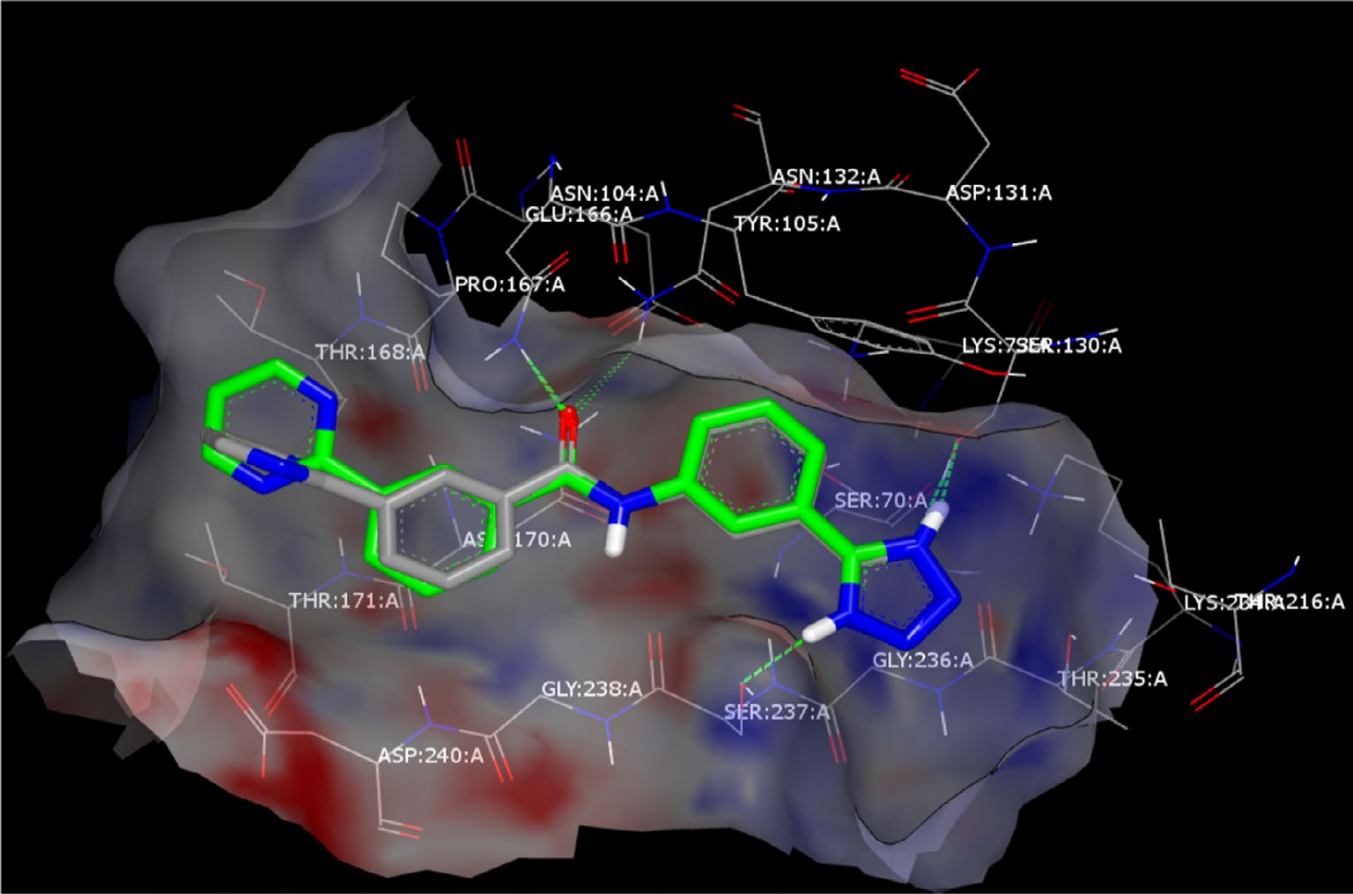
Superimposition of the docking pose between both the cocrystalized (Green) ligand and the docked reference ligand (Gray) in *β*‐lactamase enzyme.

The validation of the docking software OpenEye Hybrid for docking target ligands is carried out using DockRMSD web server. The root mean square deviation (RMSD) values between the co‐crystalized ligand (PDB:0 J7) and docked reference ligand are commonly employed to validate the accuracy of the docking simulation in predicting a closely aligned docked pose. A RMSD value of < 2 Å is considered satisfactory (Castro‐Alvarez et al. 2017). The results showed RMSD value of 1.170 Å, which is considered acceptable and showed superimposed pose between both the co‐crystalized ligand and the docked reference ligand (Figure 9).

**FIGURE 10:**
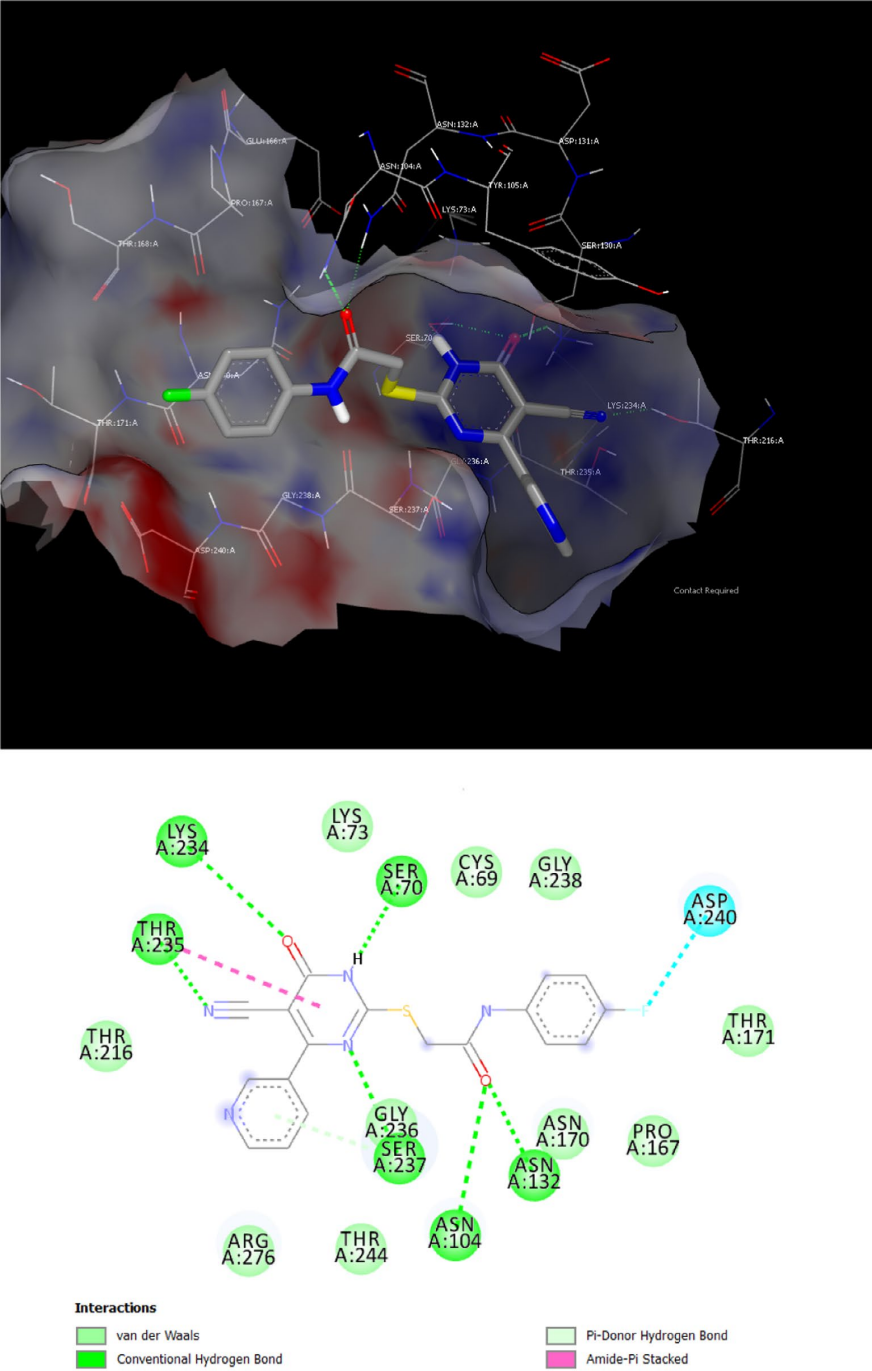
2D & 3D docking poses of derivative 3d (Blue) in *β*‐lactamases binding site.

**FIGURE 11:**
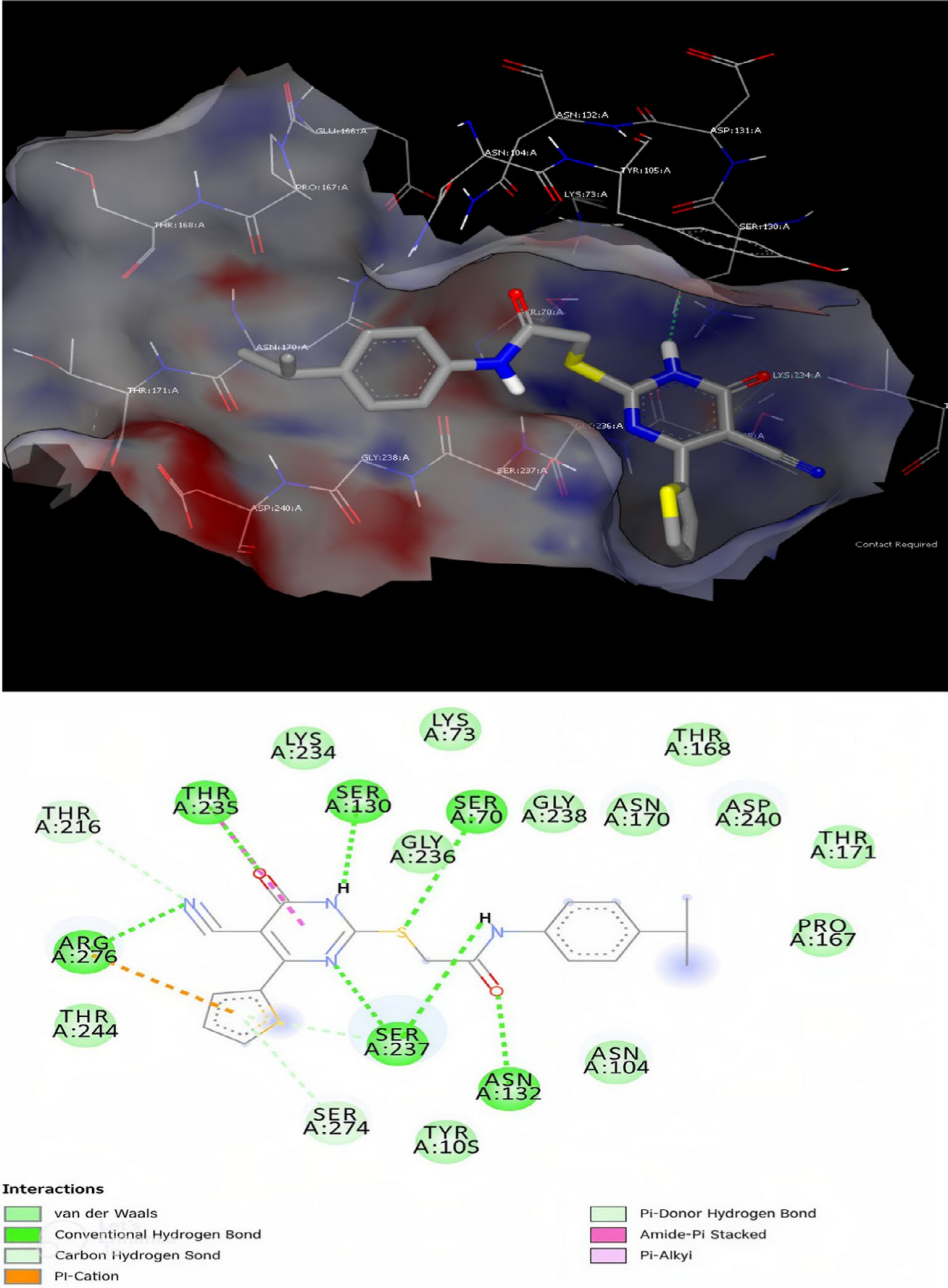
2D & 3D docking poses of derivative 3i (Blue) in *β*‐lactamases binding site.

**FIGURE 12:**
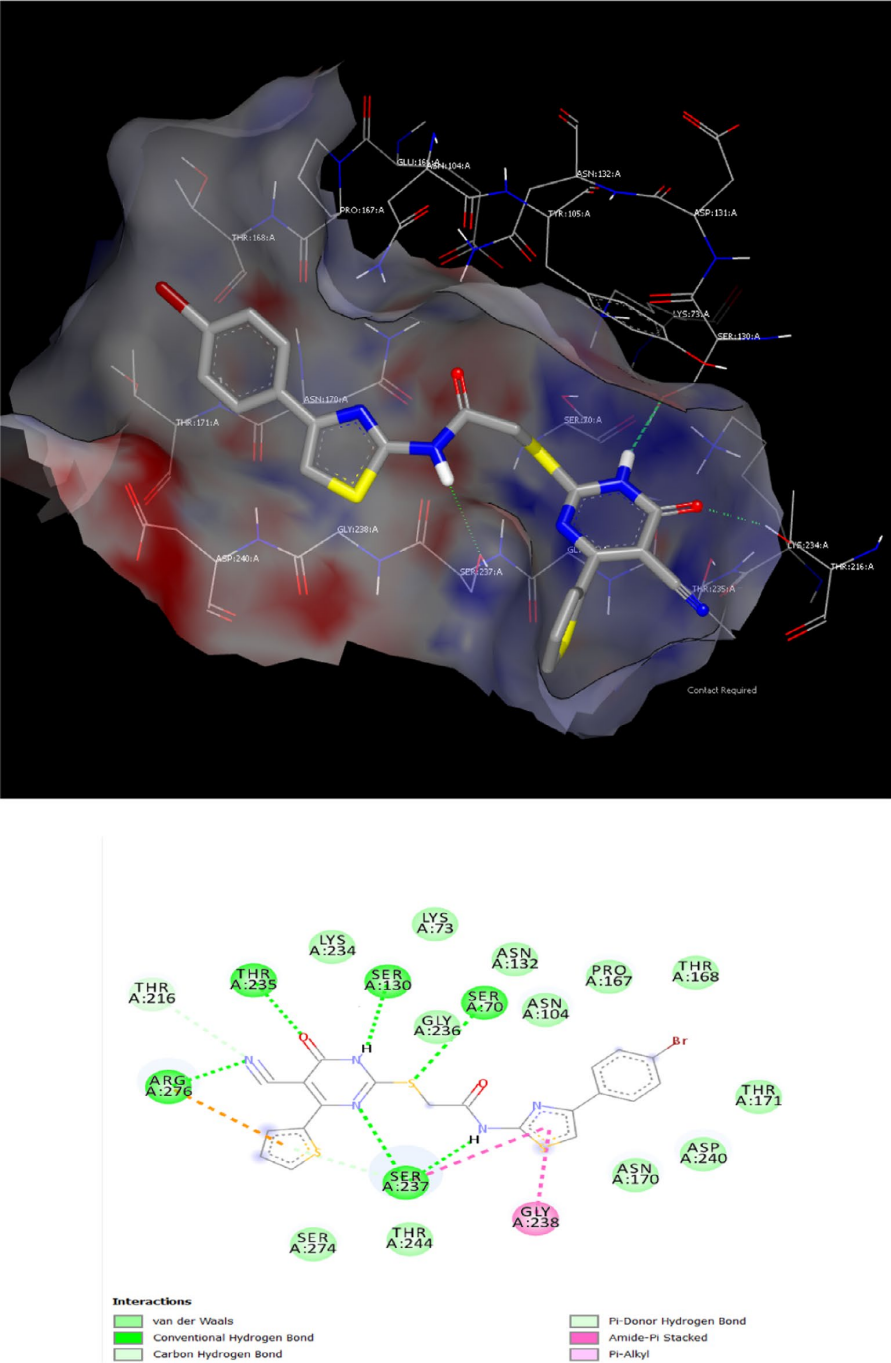
2D & 3D docking poses of derivative 7b (Blue) in *β*‐lactamases binding site.

Molecular docking simulations were conducted on the target compounds against the binding site of the protein (PDB: 4DDS). Based on their preliminary biological studies, compounds **3d**, **3i**, and **7b** were prioritized for detailed docking analysis and compared against the reference ligand (Table 5). The computational results (Figures 10, 11, 12) revealed binding interactions and identified specific hotspots within the active site.

**TABLE 5:** Comparison of binding hotspot interactions between target compounds and reference ligand.

Binding interactions	3d	3i	7b	Co‐crystalized ligand
Ser70	+Ve	+Ve	+Ve	+Ve
Asn104	−Ve	−Ve	−Ve	+Ve
Ser130	−Ve	+Ve	+Ve	+Ve
Asn132	+Ve	+Ve	−Ve	+Ve
Pro167	−Ve	−Ve	−Ve	+Ve
Thr235	+Ve	+Ve	+Ve	+Ve
Ser237	+Ve	+Ve	+Ve	+Ve
Asp240	+Ve	−Ve	−Ve	+Ve
Arg276	−Ve	+Ve	+Ve	−Ve
Thr216	−Ve	+Ve	+Ve	−Ve

All three compounds formed hydrogen bond interactions with the residues Thr235, Ser237, and Asp240. Furthermore, unlike the reference drug, these derivatives effectively anchored within an unoccupied hydrophobic pocket through interactions with Arg276. Site‐specific analysis indicated that **3d** established a halogen‐mediated hydrophobic interaction with Asp240, **3i** exhibited a hydrophobic interaction with Thr235, and finally, the thiazole moiety of **7b** formed a hydrophobic interaction with Gly238.

The binding affinities of the reference ligand and target compounds **3d, 3i**, and **7b** are as seen in (Table 6). The binding affinity of **3d**, **3i**, and **7b** was lower than that of the reference ligand. However, this difference in binding affinity may be attributed to the hydrophobic interactions in the newly identified pocket (Motiejunas and Wade 2007).

**TABLE 6:** Binding affinities of promising target compounds to reference ligand.

Compound	Docking score (kcal/mol)
**7b**	−10.6999
**3i**	−9.0740
**3d**	−7.2998
Reference ligand (0J7)	−12.0096

#### 3.3.2 Toxicity Prediction

Toxicity screening using the online web application pkCSM is described as the best target compounds in the table below (Pires et al. 2015; Table 7).

**TABLE 7:** Toxicity screening for target compounds.

Model name	Unit	3d	3i	7b
AMES toxicity	Categorical (Yes/No)	No	No	No
Max. tolerated dose (human)	Numeric (log mg/kg/day) below 0.477	0.19	0.367	0.33
hERG I inhibitor	Categorical (Yes/No)	No	No	No
hERG II inhibitor	Categorical (Yes/No)	No	No	No
Skin sensitization	Categorical (Yes/No)	No	No	No
Minnow toxicity	Numeric (log mM) Below 0.5 mM	0.655	0.379	0.379

*Note:* AMES toxicity: *Salmonella typhimurium* reverse mutation assay (Mortelmans and Zeiger 2000). hERG I and II inhibitor: Predictor of cardiac side effects (Zhang et al. 2016). Minnoow Toxicity: LC_50_ lethal concentration that causes 50% death of Fathead minnow (Ankley and Villeneuve 2006).

The findings suggest that the target compounds have no mutagenic, cardiac, skin, and Minnow toxicity. Furthermore, low maximum tolerated dosage in humans.

## 4 Conclusion

A series of substituted 1,6‐dihydropyrimidinone derivatives was designed and synthesized, followed by a comprehensive evaluation of their biological profiles to determine their efficacy as antibacterial agents. All compounds exhibited antibacterial activity using the Agar Diffusion method. Selectedtarget compounds showed potentiation effect with selected antibiotics against *Staphylococcus aureus, Bacillus subtilis* as a gram‐positive species whereas *Pseudomonas aeruginosa* as a gram‐negative species. Three compounds **3d, 3i**, **and 7b** exhibited comparable activity against *β*‐lactamase enzyme (IC50 = 0.758, 0.400, and 0.524 nM), respectively compared to clavulanic acid (IC50 = 0.934 nM). “The results of the molecular docking study suggest that the binding mode of the tested compounds is compatible with the CTX‐M‐9 class A *β*‐lactamase binding site.” The computational analysis of the physicochemical and pharmacokinetic characteristics of the newly synthesized compounds showed their potential biological efficacy and promising pharmacokinetic features. Compounds **3d, 3i**, **and 7b** could be considered as useful leads for future development to obtain more *β*‐lactamase inhibitors.

## Author Contributions

**Ahmed M. Soliman:** investigation, writing – original draft, methodology, validation, visualization, writing – review and editing, software, project administration, formal analysis, data curation, resources, funding acquisition. **Walaa R. Mahmoud:** conceptualization, supervision, investigation, methodology, validation, visualization, writing – review and editing, formal analysis, project administration, data curation, resources, writing – original draft. **Bassem H. Naguib:** conceptualization, investigation, funding acquisition, writing – original draft, validation, methodology, visualization, writing – review and editing, formal analysis, project administration, data curation, supervision, resources. **Heba Abdelrasheed Allam:** conceptualization, supervision, resources, data curation, project administration, methodology, validation, visualization, writing – review and editing, writing – original draft, investigation, formal analysis.

## Funding

The authors have nothing to report.

## Conflicts of Interest

The authors declare no conflicts of interest.

## Supporting information


**Figure S1:** HNMR spectra for compound **3a**.
**Figure S2:** CNMR spectra for compound **3a**.
**Figure S3:** HNMR spectra for compound **3b**.
**Figure S4:** CNMR spectra for compound **3b**.
**Figure S5:** HNMR spectra for compound **3c**.
**Figure S6:** CNMR spectra for compound **3c**.
**Figure S7:** HNMR spectra for compound **3d**.
**Figure S8:** CNMR spectra for compound **3d**.
**Figure S9:** HNMR spectra for compound **3e**.
**Figure S10:** CNMR spectra for compound **3e**.
**Figure S11:** HNMR spectra for compound **3f**.
**Figure S12:** CNMR spectra for compound **3f**.
**Figure S13:** HNMR spectra for compound **3g**.
**Figure S14:** CNMR spectra for compound **3g**.
**Figure S15:** HNMR spectra for compound **3h**.
**Figure S16:** CNMR spectra for compound **3h**.
**Figure S17:** HNMR spectra for compound **3i**.
**Figure S18:** CNMR spectra for compound **3i**.
**Figure S19:** HNMR spectra for compound **3j**.
**Figure S20:** CNMR spectra for compound **3j**.
**Figure S21:** HNMR spectra for compound **3k**.
**Figure S22:** CNMR spectra for compound **3k**.
**Figure S23:** HNMR spectra for compound **3l**.
**Figure S24:** CNMR spectra for compound **3l**.
**Figure S25:** HNMR spectra for compound **5a**.
**Figure S26:** CNMR spectra for compound **5a**.
**Figure S27:** HNMR spectra for compound **5b**.
**Figure S28:** CNMR spectra for compound **5b**.
**Figure S29:** HNMR spectra for compound **5c**.
**Figure S30:** CNMR spectra for compound **5c**.
**Figure S31:** HNMR spectra for compound **5d**.
**Figure S32:** CNMR spectra for compound **5d**.
**Figure S33:** HNMR spectra for compound **7a**.
**Figure S34:** CNMR spectra for compound **7a**.
**Figure S35:** HNMR spectra for compound **7b**.
**Figure S36:** CNMR spectra for compound **7b**.
**Figure S39:** Results of *β‐*Lactamase enzyme activity inhibition.
**Table S1:** Table of the *β*‐Lactamase enzyme activity inhibition.

## Data Availability

The data supporting the findings of this study are available within the article and/or its Supporting Information S1.
