# Supplementary material for: Rational Design, Synthesis, and Biological Evaluation of New Substituted Dihydropyrimidinones as Antibacterial Agents and β‐Lactamase Inhibitors
Source: Chem Biol Drug Des. 2026 Aug 4;108(2):e70374. doi: 10.1111/cbdd.70374 (PMC13438106; doi:10.1111/cbdd.70374)
Supplement: Supplementary file 1 [file CBDD-108-e70374-s001.docx]

**Supporting Information: Copies of NMR spectra for compounds 3(a-l), 5(a-d) and 7(a,b). *In-vitro*** **antibacterial results (The agar well diffusion method) , *In-vitro*** ***β*-Lactamase enzyme assay (The agar disk diffusion for** ***β*-Lactamase resistant strains) and *In-vitro*** ***β*-Lactamase enzyme assay.**

**Rational Design, Synthesis and Biological Evaluation of New Substituted dihydropyrimidinone as antibacterial agents and *β-*lactamase inhibitors.**

Ahmed M.Soliman^a^, Heba A.Allam^b^, Walaa R. Mahmoud^b^, Bassem H. Naguib^a^

*^a^ Department of Pharmaceutical Chemistry, Faculty of Pharmacy, The British University in Egypt, El-Sherouk City, Cairo 11837, Egypt*

*^b^Department of pharmaceutical chemistry, Faculty of pharmacy, Cairo University, Cairo, Egypt.*

**Table of contents.**

| **NMR spectra for compounds 3(a-l), 5(a-d) and 7(a,b).** | **Pages 2-37** | **Figures S1-36** |
| --- | --- | --- |
| ***In-vitro*** **antibacterial results (The agar well diffusion method)** | **Pages 38-39** | **Figure S37** |
| ***In-vitro* *β*-Lactamase enzyme assay (The agar disk diffusion for *β*-Lactamase resistant strains)** | **Pages 40-41** | **Figure S38** |
| ***In-vitro*** ***β*-Lactamase enzyme assay.** | **Pages 42-49** | **Table S1 – Figure S39** |

1) **NMR spectra for Target compounds**

|  | |
| --- | --- |
|  |  |
| **Figure S1:** **HNMR spectra for compound 3a.** | |

**Figure S2:** **CNMR spectra for compound 3a.**

|  | |
| --- | --- |
|  |  |
| **Figure S3:** **HNMR spectra for compound 3b.** | |

**Figure S4:** **CNMR spectra for compound 3b.**

|  | |
| --- | --- |
|  |  |
| **Figure S5:** **HNMR spectra for compound 3c.** | |

**Figure S6:** **CNMR spectra for compound 3c.**

**Figure S7:** **HNMR spectra for compound 3d**

**Figure S8:** **CNMR spectra for compound 3d.**

**Figure S9:** **HNMR spectra for compound 3e.**

**Figure S10:** **CNMR spectra for compound 3e.**

**Figure S11:** **HNMR spectra for compound 3f.**

**Figure S12:** **CNMR spectra for compound 3f.**

|  | |
| --- | --- |
|  |  |
| **Figure S13:** **HNMR spectra for compound 3g.** | |

**Figure S14:** **CNMR spectra for compound 3g.**

**Figure S15:** **HNMR spectra for compound 3h.**

**Figure S16:** **CNMR spectra for compound 3h.**

**Figure S17:** **HNMR spectra for compound 3i.**

**Figure S18:** **CNMR spectra for compound 3i.**

**Figure S19:** **HNMR spectra for compound 3j.**

**Figure S20:** **CNMR spectra for compound 3j.**

|  | |
| --- | --- |
|  |  |
| **Figure S21:** **HNMR spectra for compound 3k.** | |

**Figure S22:** **CNMR spectra for compound 3k.**

**Figure S23:** **HNMR spectra for compound 3l.**

**Figure S24:** **CNMR spectra for compound 3l.**

|  | |
| --- | --- |
|  |  |
| **Figure S25:** **HNMR spectra for compound 5a.** | |

**Figure S26:** **CNMR spectra for compound 5a.**

**Figure S27:** **HNMR spectra for compound 5b.**

**Figure S28:** **CNMR spectra for compound 5b.**

|  | |
| --- | --- |
|  |  |
| **Figure S29:** **HNMR spectra for compound 5c.** | |

**Figure S30:** **CNMR spectra for compound 5c.**

**Figure S31:** **HNMR spectra for compound 5d.**

**Figure S32:** **CNMR spectra for compound 5d.**

|  | |
| --- | --- |
|  |  |
| **Figure S33:** **HNMR spectra for compound 7a.** | |

**Figure S34:** **CNMR spectra for compound 7a.**

|  | |
| --- | --- |
|  |  |
| **Figure S35:** **HNMR spectra for compound 7b.** | |

**Figure S36:** **CNMR spectra for compound 7b.**

| 2) ***In-vitro*** **antibacterial results (The agar well diffusion method)** | |
| --- | --- |
|  |  |
|  |  |
|  |  |
|  |  |
| **S37: snapshots of antibacterial acitvity** | |

| 3) **The agar disk diffusion for *β*-Lactamase resistant strains** | |
| --- | --- |
|  |  |
|  |  |
|  |  |
|  |  |
|  |  |
| **S38: snapshots of potentiation effect of target compounds** | |

**β -Lactamase Enzyme Assay**

**Type of analysis:** ***β*-lactamase inhibition assay**

**Tested Cell line: No cell lines used or any biological homogenate, the target compound activity tested against the chemical enzyme Kit.**

**Reader: Tecan spark Multimode Plate Reader (ab197009 Beta Lactamase Inhibitor Screening Assay Kit (Colorimetric))**

**Solvent: DMSO**

**No of samples: 18 samples**

**Results:**

**Table S1: Table of the *β*-Lactamase enzyme activity inhibition:**

| **Compound** | **β -Lactamase Conc (IC_50_)** | | | |
| --- | --- | --- | --- | --- |
| **Code** | **MW**  **(g/mol)** | **IC_50_**  **(µg/ml)** | **IC_50_**  **(µmol/ml)** | **IC_50_ (nM) ≡**  **(nmol/ml)** |
| **3a** | **363.4** | **0.686** | **0.00188773** | **1.88772702 ± 0.069** |
| **3b** | **377.42** | **1.973** | **0.0052276** | **5.2275979 ± 0.191** |
| **3c** | **405.48** | **0.633** | **0.00156111** | **1.56111276 ± 0.057** |
| **3d** | **381.39** | **0.289** | **0.00075775** | **0.75775453 ± 0.029** |
| **3e** | **431.28** | **1.625** | **0.00376785** | **3.76785383 ± 0.139** |
| **3f** | **397.84** | **7.807** | **0.01962347** | **19.6234667 ± 0.719** |
| **3g** | **368.43** | **2.007** | **0.00544744** | **5.44743913 ± 0.201** |
| **3h** | **382.46** | **1.147** | **0.00299901** | **2.99900643 ± 0.110** |
| **3i** | **410.51** | **0.164** | **0.0003995** | **0.39950306 ± 0.015** |
| **3j** | **386.42** | **0.406** | **0.00105067** | **1.05067026 ± 0.039** |
| **3k** | **435.31** | **2.334** | **0.0053617** | **5.36169626 ± 0.198** |
| **3(l )** | **402.87** | **0.792** | **0.00196589** | **1.96589471 ± 0.072** |
| **5a** | **362.41** | **1.797** | **0.00495847** | **4.95847245 ± 0.080** |
| **5b** | **427.28** | **0.605** | **0.00141593** | **1.41593335 ± 0.042** |
| **5c** | **367.44** | **0.405** | **0.00110222** | **1.10222077 ± 0.038** |
| **5d** | **432.31** | **3.862** | **0.0089334** | **8.93340427 ± 0.671** |
| **7a** | **460.53** | **1.225** | **0.00265998** | **2.65997872 ± 0.413** |
| **7b** | **530.43** | **0.278** | **0.0005241** | **0.52410309 ± 0.040** |
| **Clavulanic acid** | **199.16** | **0.186** | **0.00093392** | **0.93392247 ± 0.035** |

**Figure S39: Results of *β-*Lactamase enzyme activity inhibition.**

Fully detailed results:

| **β -Lactamase enzyme activity** | | | | | | | | | | | | | | | | | | | | |
| --- | --- | --- | --- | --- | --- | --- | --- | --- | --- | --- | --- | --- | --- | --- | --- | --- | --- | --- | --- | --- |
| **Codea** | | **conc** | **Log Conc.** | | **%inh** | | **T2** | | **T1** | | **∆T** | | **RFU2** | | **RFU1** | | **∆RFU** | **slope** | **K.**  **Activity** | **IC50**  **(µg/ml)** |
| **3a** | | 100 | 2 | | 91.4 | | 30 | | 0 | | 30 | | 8.64 | | 0 | | 8.64 | 3.333 | 10.368 | **0.686**  **±**0.025 |
|  |  | 10 | 1 | | 76.9 | | 30 | | 0 | | 30 | | 23.07 | | 0 | | 23.07 | 3.333 | 27.684 |  |
|  |  | 1 | 0 | | 51.1 | | 30 | | 0 | | 30 | | 48.93 | | 0 | | 48.93 | 3.333 | 58.716 |  |
|  |  | 0.1 | -1 | | 31.8 | | 30 | | 0 | | 30 | | 68.17 | | 0 | | 68.17 | 3.333 | 81.804 |  |
|  |  | 0.01 | -2 | | 15 | | 30 | | 0 | | 30 | | 85.01 | | 0 | | 85.01 | 3.333 | 102.01 |  |
| **EC(contro)** | | **-** | **-** | | **0** | | **30** | | **0** | | **30** | | **100** | | **0** | | **100** | **3.333** | **120** |  |
|  | |  |  | |  | |  | |  | |  | |  | |  | |  |  |  |  |
| **Code** | | **conc** | **log** | | **%inh** | | **T2** | | **T1** | | **∆T** | | **RFU2** | | **RFU1** | | **∆RFU** | **slope** | **K.**  **Activity** | **1.973**  **±0.072** |
| **3b** | | 100 | 2 | | 88.5 | | 30 | | 0 | | 30 | | 11.54 | | 0 | | 11.54 | 3.333 | 13.848 |  |
|  |  | 10 | 1 | | 70.2 | | 30 | | 0 | | 30 | | 29.77 | | 0 | | 29.77 | 3.333 | 35.724 |  |
|  |  | 1 | 0 | | 37.6 | | 30 | | 0 | | 30 | | 62.39 | | 0 | | 62.39 | 3.333 | 74.868 |  |
|  |  | 0.1 | -1 | | 15.9 | | 30 | | 0 | | 30 | | 84.13 | | 0 | | 84.13 | 3.333 | 100.96 |  |
|  |  | 0.01 | -2 | | 5.24 | | 30 | | 0 | | 30 | | 94.76 | | 0 | | 94.76 | 3.333 | 113.71 |  |
| **EC(contro)** | | **-** | **-** | | **0** | | **30** | | **0** | | **30** | | **100** | | **0** | | **100** | **3.333** | **120** |  |
|  | |  |  | |  | |  | |  | |  | |  | |  | |  |  |  |  |
| **Code** | | **conc** | **log** | | **%inh** | | **T2** | | **T1** | | **∆T** | | **RFU2** | | **RFU1** | | **∆RFU** | **slope** | **K.**  **Activity** | **0.633**  **±0.023** |
| **3c** | | 100 | 2 | | 94.3 | | 30 | | 0 | | 30 | | 5.71 | | 0 | | 5.71 | 3.333 | 6.852 |  |
|  |  | 10 | 1 | | 83.5 | | 30 | | 0 | | 30 | | 16.45 | | 0 | | 16.45 | 3.333 | 19.74 |  |
|  |  | 1 | 0 | | 51.7 | | 30 | | 0 | | 30 | | 48.33 | | 0 | | 48.33 | 3.333 | 57.996 |  |
|  |  | 0.1 | -1 | | 30.2 | | 30 | | 0 | | 30 | | 69.75 | | 0 | | 69.75 | 3.333 | 83.7 |  |
|  |  | 0.01 | -2 | | 11.9 | | 30 | | 0 | | 30 | | 88.12 | | 0 | | 88.12 | 3.333 | 105.74 |  |
| **EC(contro)** | | **-** | **-** | | **0** | | **30** | | **0** | | **30** | | **100** | | **0** | | **100** | **3.333** | **120** |  |
|  | |  |  | |  | |  | |  | |  | |  | |  | |  |  |  |  |
| **Code** | | **conc** | **log** | | **%inh** | | **T2** | | **T1** | | **∆T** | | **RFU2** | | **RFU1** | | **∆RFU** | **slope** | **K.**  **Activity** | **0.289**  **±0.011** |
| **3d** | | 100 | 2 | | 93.1 | | 30 | | 0 | | 30 | | 6.86 | | 0 | | 6.86 | 3.333 | 8.232 |  |
|  |  | 10 | 1 | | 80.6 | | 30 | | 0 | | 30 | | 19.42 | | 0 | | 19.42 | 3.333 | 23.304 |  |
|  |  | 1 | 0 | | 64.1 | | 30 | | 0 | | 30 | | 35.88 | | 0 | | 35.88 | 3.333 | 43.056 |  |
|  |  | 0.1 | -1 | | 41.2 | | 30 | | 0 | | 30 | | 58.76 | | 0 | | 58.76 | 3.333 | 70.512 |  |
|  |  | 0.01 | -2 | | 20.6 | | 30 | | 0 | | 30 | | 79.41 | | 0 | | 79.41 | 3.333 | 95.292 |  |
| **EC(contro)** | | **-** | **-** | | **0** | | **30** | | **0** | | **30** | | **100** | | **0** | | **100** | **3.333** | **120** |  |
|  | |  |  | |  | |  | |  | |  | |  | |  | |  |  |  |  |
| **Code** | | **conc** | **log** | | **%inh** | | **T2** | | **T1** | | **∆T** | | **RFU2** | | **RFU1** | | **∆RFU** | **slope** | **K.**  **Activity** | **1.625**  **±0.06** |
| **3e** | | 100 | 2 | | 92.4 | | 30 | | 0 | | 30 | | 7.59 | | 0 | | 7.59 | 3.333 | 9.108 |  |
|  |  | 10 | 1 | | 78.7 | | 30 | | 0 | | 30 | | 21.33 | | 0 | | 21.33 | 3.333 | 25.596 |  |
|  |  | 1 | 0 | | 36.1 | | 30 | | 0 | | 30 | | 63.88 | | 0 | | 63.88 | 3.333 | 76.656 |  |
|  |  | 0.1 | -1 | | 13.5 | | 30 | | 0 | | 30 | | 86.49 | | 0 | | 86.49 | 3.333 | 103.79 |  |
|  |  | 0.01 | -2 | | 3.74 | | 30 | | 0 | | 30 | | 96.26 | | 0 | | 96.26 | 3.333 | 115.51 |  |
| **EC(contro)** | | **-** | **-** | | **0** | | **30** | | **0** | | **30** | | **100** | | **0** | | **100** | **3.333** | **120** |  |
|  | |  |  | |  | |  | |  | |  | |  | |  | |  |  |  |  |
| **Code** | | **conc** | **log** | | **%inh** | | **T2** | | **T1** | | **∆T** | | **RFU2** | | **RFU1** | | **∆RFU** | **slope** | **K.**  **Activity** | **7.807**  **±0.286** |
| **3f** | | 100 | 2 | | 81.6 | | 30 | | 0 | | 30 | | 18.39 | | 0 | | 18.39 | 3.333 | 22.068 |  |
|  |  | 10 | 1 | | 57.9 | | 30 | | 0 | | 30 | | 42.09 | | 0 | | 42.09 | 3.333 | 50.508 |  |
|  |  | 1 | 0 | | 8.56 | | 30 | | 0 | | 30 | | 91.44 | | 0 | | 91.44 | 3.333 | 109.73 |  |
|  |  | 0.1 | -1 | | 5.38 | | 30 | | 0 | | 30 | | 94.62 | | 0 | | 94.62 | 3.333 | 113.54 |  |
|  |  | 0.01 | -2 | | 2.42 | | 30 | | 0 | | 30 | | 97.58 | | 0 | | 97.58 | 3.333 | 117.1 |  |
| **EC(contro)** | | **-** | **-** | | **0** | | **30** | | **0** | | **30** | | **100** | | **0** | | **100** | **3.333** | **120** |  |
|  | |  |  | |  | |  | |  | |  | |  | |  | |  |  |  |  |
| **Code** | | **conc** | **log** | | **%inh** | | **T2** | | **T1** | | **∆T** | | **RFU2** | | **RFU1** | | **∆RFU** | **slope** | **K.**  **Activity** | **2.007**  **±0.074** |
| **3g** | | 100 | 2 | | 87.9 | | 30 | | 0 | | 30 | | 12.06 | | 0 | | 12.06 | 3.333 | 14.472 |  |
|  |  | 10 | 1 | | 70.2 | | 30 | | 0 | | 30 | | 29.77 | | 0 | | 29.77 | 3.333 | 35.724 |  |
|  |  | 1 | 0 | | 41.1 | | 30 | | 0 | | 30 | | 58.93 | | 0 | | 58.93 | 3.333 | 70.716 |  |
|  |  | 0.1 | -1 | | 12.9 | | 30 | | 0 | | 30 | | 87.11 | | 0 | | 87.11 | 3.333 | 104.53 |  |
|  |  | 0.01 | -2 | | 3.73 | | 30 | | 0 | | 30 | | 96.27 | | 0 | | 96.27 | 3.333 | 115.52 |  |
| **EC(contro)** | | **-** | **-** | | **0** | | **30** | | **0** | | **30** | | **100** | | **0** | | **100** | **3.333** | **120** |  |
|  | |  |  | |  | |  | |  | |  | |  | |  | |  |  |  |  |
| **Code** | | **conc** | **log** | | **%inh** | | **T2** | | **T1** | | **∆T** | | **RFU2** | | **RFU1** | | **∆RFU** | **slope** | **K.**  **Activity** | **1.147 ±0.042** |
| **3h** | | 100 | 2 | | 92.1 | | 30 | | 0 | | 30 | | 7.91 | | 0 | | 7.91 | 3.333 | 9.492 |  |
|  |  | 10 | 1 | | 78.3 | | 30 | | 0 | | 30 | | 21.72 | | 0 | | 21.72 | 3.333 | 26.064 |  |
|  |  | 1 | 0 | | 44.6 | | 30 | | 0 | | 30 | | 55.41 | | 0 | | 55.41 | 3.333 | 66.492 |  |
|  |  | 0.1 | -1 | | 20.9 | | 30 | | 0 | | 30 | | 79.06 | | 0 | | 79.06 | 3.333 | 94.872 |  |
|  |  | 0.01 | -2 | | 7.33 | | 30 | | 0 | | 30 | | 92.67 | | 0 | | 92.67 | 3.333 | 111.2 |  |
| **EC(contro)** | | **-** | **-** | | **0** | | **30** | | **0** | | **30** | | **100** | | **0** | | **100** | **3.333** | **120** |  |
|  | |  |  | |  | |  | |  | |  | |  | |  | |  |  |  |  |
| **Code** | | **conc** | **log** | | **%inh** | | **T2** | | **T1** | | **∆T** | | **RFU2** | | **RFU1** | | **∆RFU** | **slope** | **K.**  **Activity** | **0.164**  **±0.006** |
| **3i** | | 100 | 2 | | 95.7 | | 30 | | 0 | | 30 | | 4.28 | | 0 | | 4.28 | 3.333 | 10.848 |  |
|  |  | 10 | 1 | | 86.9 | | 30 | | 0 | | 30 | | 13.06 | | 0 | | 13.06 | 3.333 | 21.456 |  |
|  |  | 1 | 0 | | 70.6 | | 30 | | 0 | | 30 | | 29.44 | | 0 | | 29.44 | 3.333 | 42.732 |  |
|  |  | 0.1 | -1 | | 42.7 | | 30 | | 0 | | 30 | | 57.29 | | 0 | | 57.29 | 3.333 | 75.312 |  |
|  |  | 0.01 | -2 | | 26.1 | | 30 | | 0 | | 30 | | 73.94 | | 0 | | 73.94 | 3.333 | 104.8 |  |
| **EC(contro)** | | **-** | **-** | | **0** | | **30** | | **0** | | **30** | | **100** | | **0** | | **100** | **3.333** | **120** |  |
|  | |  |  | |  | |  | |  | |  | |  | |  | |  |  |  |  |
| **Code** | | **conc** | **log** | | **%inh** | | **T2** | | **T1** | | **∆T** | | **RFU2** | | **RFU1** | | **∆RFU** | **slope** | **K.**  **Activity** | **0.406**  **±0.015** |
| **3j** | | 100 | 2 | | 94.3 | | 30 | | 0 | | 30 | | 5.69 | | 0 | | 5.69 | 3.333 | 6.828 |  |
|  |  | 10 | 1 | | 84.2 | | 30 | | 0 | | 30 | | 15.77 | | 0 | | 15.77 | 3.333 | 18.924 |  |
|  |  | 1 | 0 | | 61.6 | | 30 | | 0 | | 30 | | 38.41 | | 0 | | 38.41 | 3.333 | 46.092 |  |
|  |  | 0.1 | -1 | | 37.2 | | 30 | | 0 | | 30 | | 62.83 | | 0 | | 62.83 | 3.333 | 75.396 |  |
|  |  | 0.01 | -2 | | 13.5 | | 30 | | 0 | | 30 | | 86.49 | | 0 | | 86.49 | 3.333 | 103.79 |  |
| **EC(contro)** | | **-** | **-** | | **0** | | **30** | | **0** | | **30** | | **100** | | **0** | | **100** | **3.333** | **120** |  |
|  | |  |  | |  | |  | |  | |  | |  | |  | |  |  |  |  |
| **Code** | | **conc** | **log** | | **%inh** | | **T2** | | **T1** | | **∆T** | | **RFU2** | | **RFU1** | | **∆RFU** | **slope** | **K.**  **Activity** | **2.334**  **±0.086** |
| **3k** | | 100 | 2 | | 90.6 | | 30 | | 0 | | 30 | | 9.39 | | 0 | | 9.39 | 3.333 | 11.268 |  |
|  |  | 10 | 1 | | 72.6 | | 30 | | 0 | | 30 | | 27.41 | | 0 | | 27.41 | 3.333 | 32.892 |  |
|  |  | 1 | 0 | | 30.1 | | 30 | | 0 | | 30 | | 69.88 | | 0 | | 69.88 | 3.333 | 83.856 |  |
|  |  | 0.1 | -1 | | 8.94 | | 30 | | 0 | | 30 | | 91.06 | | 0 | | 91.06 | 3.333 | 109.27 |  |
|  |  | 0.01 | -2 | | 4.22 | | 30 | | 0 | | 30 | | 95.78 | | 0 | | 95.78 | 3.333 | 114.94 |  |
| **EC(contro)** | | **-** | **-** | | **0** | | **30** | | **0** | | **30** | | **100** | | **0** | | **100** | **3.333** | **120** |  |
|  | |  |  | |  | |  | |  | |  | |  | |  | |  |  |  |  |
| **Code** | | **conc** | **log** | | **%inh** | | **T2** | | **T1** | | **∆T** | | **RFU2** | | **RFU1** | | **∆RFU** | **slope** | **K.**  **Activity** | **0.792**  **±0.029** |
| **3l** | | 100 | 2 | | 92.5 | | 30 | | 0 | | 30 | | 7.52 | | 0 | | 7.52 | 3.333 | 9.024 |  |
|  |  | 10 | 1 | | 80.2 | | 30 | | 0 | | 30 | | 19.79 | | 0 | | 19.79 | 3.333 | 23.748 |  |
|  |  | 1 | 0 | | 55 | | 30 | | 0 | | 30 | | 45.02 | | 0 | | 45.02 | 3.333 | 54.024 |  |
|  |  | 0.1 | -1 | | 26.1 | | 30 | | 0 | | 30 | | 73.85 | | 0 | | 73.85 | 3.333 | 88.62 |  |
|  |  | 0.01 | -2 | | 7.53 | | 30 | | 0 | | 30 | | 92.47 | | 0 | | 92.47 | 3.333 | 110.96 |  |
| **EC(contro)** | | **-** | **-** | | **0** | | **30** | | **0** | | **30** | | **100** | | **0** | | **100** | **3.333** | **120** |  |
|  | |  |  | |  | |  | |  | |  | |  | |  | |  |  |  |  |
| **code** | **conc** | | | **Log Conc.** | | **%inh** | | **T2** | | **T1** | | **∆T** | | **RFU2** | | **RFU1** | **∆RFU** | **slope** | **K.**  **Activity** | **IC50**  **(µg/ml)** |
| **5a** | 100 | | | 2 | | 89.5 | | 30 | | 0 | | 30 | | 0.279 | | 0 | 0.279 | 3.333 | 12.5865 | **1.797**  **±0.029** |
|  | 10 | | | 1 | | 64.8 | | 30 | | 0 | | 30 | | 0.935 | | 0 | 0.935 | 3.333 | 42.18 |  |
|  | 1 | | | 0 | | 44.9 | | 30 | | 0 | | 30 | | 1.465 | | 0 | 1.465 | 3.333 | 66.09 |  |
|  | 0.1 | | | -1 | | 22 | | 30 | | 0 | | 30 | | 2.075 | | 0 | 2.075 | 3.333 | 93.609 |  |
|  | 0.01 | | | -2 | | 0.64 | | 30 | | 0 | | 30 | | 2.643 | | 0 | 2.643 | 3.333 | 119.232 |  |
| **EC(contro)** | **-** | | | **-** | | **0** | | **30** | | **0** | | **30** | | **100** | | **0** | **100** | **3.333** | **120** |  |
|  |  | | |  | |  | |  | |  | |  | |  | |  |  |  |  |  |
| **code** | **conc** | | | **log** | | **%inh** | | **T2** | | **T1** | | **∆T** | | **RFU2** | | **RFU1** | **∆RFU** | **slope** | **K.**  **Activity** | **0.605**  **±0.018** |
| **5b** | 100 | | | 2 | | 90.8 | | 30 | | 0 | | 30 | | 0.244 | | 0 | 0.244 | 3.333 | 11.0076 |  |
|  | 10 | | | 1 | | 75.6 | | 30 | | 0 | | 30 | | 0.649 | | 0 | 0.649 | 3.333 | 29.2782 |  |
|  | 1 | | | 0 | | 55.5 | | 30 | | 0 | | 30 | | 1.184 | | 0 | 1.184 | 3.333 | 53.415 |  |
|  | 0.1 | | | -1 | | 34.1 | | 30 | | 0 | | 30 | | 1.752 | | 0 | 1.752 | 3.333 | 79.038 |  |
|  | 0.01 | | | -2 | | 15 | | 30 | | 0 | | 30 | | 2.261 | | 0 | 2.261 | 3.333 | 102 |  |
| **EC(contro)** | **-** | | | **-** | | **0** | | **30** | | **0** | | **30** | | **100** | | **0** | **100** | **3.333** | **120** |  |
|  |  | | |  | |  | |  | |  | |  | |  | |  |  |  |  |  |
| **code** | **conc** | | | **log** | | **%inh** | | **T2** | | **T1** | | **∆T** | | **RFU2** | | **RFU1** | **∆RFU** | **slope** | **K.**  **Activity** | **0.405**  **±0.014** |
| **5c** | 100 | | | 2 | | 91.5 | | 30 | | 0 | | 30 | | 0.225 | | 0 | 0.225 | 3.333 | 10.1505 |  |
|  | 10 | | | 1 | | 77 | | 30 | | 0 | | 30 | | 0.613 | | 0 | 0.613 | 3.333 | 27.654 |  |
|  | 1 | | | 0 | | 57 | | 30 | | 0 | | 30 | | 1.143 | | 0 | 1.143 | 3.333 | 51.564 |  |
|  | 0.1 | | | -1 | | 40.5 | | 30 | | 0 | | 30 | | 1.582 | | 0 | 1.582 | 3.333 | 71.367 |  |
|  | 0.01 | | | -2 | | 19.4 | | 30 | | 0 | | 30 | | 2.144 | | 0 | 2.144 | 3.333 | 96.723 |  |
| **EC(contro)** | **-** | | | **-** | | **0** | | **30** | | **0** | | **30** | | **100** | | **0** | **100** | **3.333** | **120** |  |
|  |  | | |  | |  | |  | |  | |  | |  | |  |  |  |  |  |
| **code** | **conc** | | | **log** | | **%inh** | | **T2** | | **T1** | | **∆T** | | **RFU2** | | **RFU1** | **∆RFU** | **slope** | K.  Activity | **3.86**  **±0.29** |
| **5d** | 100 | | | 2 | | 85.8 | | 30 | | 0 | | 30 | | 0.377 | | 0 | 0.377 | 3.333 | 17.0076 |  |
|  | 10 | | | 1 | | 59 | | 30 | | 0 | | 30 | | 1.09 | | 0 | 1.09 | 3.333 | 49.173 |  |
|  | 1 | | | 0 | | 30.7 | | 30 | | 0 | | 30 | | 1.844 | | 0 | 1.844 | 3.333 | 83.187 |  |
|  | 0.1 | | | -1 | | 7.67 | | 30 | | 0 | | 30 | | 2.456 | | 0 | 2.456 | 3.333 | 110.796 |  |
|  | 0.01 | | | -2 | | 3.31 | | 30 | | 0 | | 30 | | 2.572 | | 0 | 2.572 | 3.333 | 116.031 |  |
| **EC(contro)** | **-** | | | **-** | | **0** | | **30** | | **0** | | **30** | | **100** | | **0** | **100** | **3.333** | **120** |  |
|  |  | | |  | |  | |  | |  | |  | |  | |  |  |  |  |  |
| **code** | **conc** | | | **log** | | **%inh** | | **T2** | | **T1** | | **∆T** | | **RFU2** | | **RFU1** | **∆RFU** | **slope** | **K.**  **Activity** | **1.22**  **±0.19** |
| **7a** | 100 | | | 2 | | 88.6 | | 30 | | 0 | | 30 | | 0.303 | | 0 | 0.303 | 3.333 | 13.6692 |  |
|  | 10 | | | 1 | | 64.6 | | 30 | | 0 | | 30 | | 0.942 | | 0 | 0.942 | 3.333 | 42.495 |  |
|  | 1 | | | 0 | | 42.4 | | 30 | | 0 | | 30 | | 1.533 | | 0 | 1.533 | 3.333 | 69.159 |  |
|  | 0.1 | | | -1 | | 28.8 | | 30 | | 0 | | 30 | | 1.894 | | 0 | 1.894 | 3.333 | 85.443 |  |
|  | 0.01 | | | -2 | | 17.8 | | 30 | | 0 | | 30 | | 2.186 | | 0 | 2.186 | 3.333 | 98.616 |  |
| **EC(contro)** | **-** | | | **-** | | **0** | | **30** | | **0** | | **30** | | **100** | | **0** | **100** | **3.333** | **120** |  |
|  |  | | |  | |  | |  | |  | |  | |  | |  |  |  |  |  |
| **code** | **conc** | | | **log** | | **%inh** | | **T2** | | **T1** | | **∆T** | | **RFU2** | | **RFU1** | **∆RFU** | **slope** | **K.**  **Activity** | **0.278**  **±0.021** |
| **7b** | 100 | | | 2 | | 90.9 | | 30 | | 0 | | 30 | | 0.241 | | 0 | 0.241 | 3.333 | 10.8723 |  |
|  | 10 | | | 1 | | 73.4 | | 30 | | 0 | | 30 | | 0.707 | | 0 | 0.707 | 3.333 | 31.896 |  |
|  | 1 | | | 0 | | 61.1 | | 30 | | 0 | | 30 | | 1.034 | | 0 | 1.034 | 3.333 | 46.647 |  |
|  | 0.1 | | | -1 | | 44.3 | | 30 | | 0 | | 30 | | 1.481 | | 0 | 1.481 | 3.333 | 66.813 |  |
|  | 0.01 | | | -2 | | 25 | | 30 | | 0 | | 30 | | 1.996 | | 0 | 1.996 | 3.333 | 90.045 |  |
| **EC(contro)** | **-** | | | **-** | | **0** | | **30** | | **0** | | **30** | | **100** | | **0** | **100** | **3.333** | **120** |  |
|  |  | | |  | |  | |  | |  | |  | |  | |  |  |  |  |  |
| **code** | **conc** | | | **log** | | **%inh** | | **T2** | | **T1** | | **∆T** | | **RFU2** | | **RFU1** | **∆RFU** | **slope** | **K.**  **Activity** | **0.186**  **±0.007** |
| **Clavulanic acid** | 100 | | | 2 | | 95 | | 30 | | 0 | | 30 | | 4.96 | | 0 | 4.96 | 3.333 | 5.952 |  |
|  | 10 | | | 1 | | 88 | | 30 | | 0 | | 30 | | 12.02 | | 0 | 12.02 | 3.333 | 14.424 |  |
|  | 1 | | | 0 | | 70.2 | | 30 | | 0 | | 30 | | 29.81 | | 0 | 29.81 | 3.333 | 35.772 |  |
|  | 0.1 | | | -1 | | 42.6 | | 30 | | 0 | | 30 | | 57.37 | | 0 | 57.37 | 3.333 | 68.844 |  |
|  | 0.01 | | | -2 | | 23.2 | | 30 | | 0 | | 30 | | 76.75 | | 0 | 76.75 | 3.333 | 92.1 |  |
| **EC(contro)** | **-** | | | **-** | | **0** | | **30** | | **0** | | **30** | | **100** | | **0** | **100** | **3.333** | **120** |  |
|  |  | | |  | |  | |  | |  | |  | |  | |  |  |  |  |  |

#EC🡪referred to Control (Kit without any testing compound) and represented full activity or 100% activity

#%Inh 🡪 referred to the inhibition percentage caused by the tested compound

\# T1 and T2 🡪 the starting and ending time and the difference (∆T=30 min.) represented the reaction time between enzyme kit and tested compound

\# RFU1 and RFU2 🡪 each one of them represented the relative fluorescence (or by the mean Optical density) unit at time T1 and T2 and the difference (∆ RFU) represents the total relative fluorescence after reaction completion (30min.)

\# K. activity 🡪 referred to Kinetic activity of the tested compound after reaction time completion

\# IC_50_ 🡪referred to Half maximal enzyme activity concentration
